# Identification of Cognitive Training for Individuals with Parkinson’s Disease: A Systematic Review

**DOI:** 10.3390/brainsci15010061

**Published:** 2025-01-11

**Authors:** Marina Francesca Gattoni, Silvia Gobbo, Sarah Feroldi, Anna Salvatore, Jorge Navarro, Sandro Sorbi, Francesca Lea Saibene

**Affiliations:** 1 IRCCS Fondazione Don Carlo Gnocchi ONLUS, 20148 Milan, Italy; 2 Department of Statistics, Informatics, Applications “G. Parenti”, University of Florence, 50134 Florence, Italy; 3 Department of Psychology, University of Milan-Bicocca, 20126 Milan, Italy; 4 IRCCS Fondazione Don Carlo Gnocchi ONLUS, 50143 Florence, Italy

**Keywords:** Parkinson’s disease, PD, cognitive training, cognitive rehabilitation, cognitive functions, cognition, systematic review

## Abstract

**Background/Objectives**: Parkinson’s disease (PD) is a neurodegenerative disorder, characterised by cardinal motor features and a multitude of non-motor manifestations. Among them, cognitive impairment in PD has been recognised as a defined clinical entity, and it might lead to an increased risk of developing dementia. Consequently, the present review aimed to ascertain the available interventions for the training of cognitive abilities in persons with PD (PwPD). **Methods**: PRISMA guidelines were followed to select studies in the following databases: PubMed, PsycINFO, and Web of Science. Two independent reviewers conducted the different phases of the review, and a third expert was called in to address any doubts/conflicts. Randomised controlled trials and randomised clinical trials concerning cognitive training with cognitive outcomes in PwPD were selected. **Results**: A total of 28 articles were included. The considered studies applied various experimental interventions for the training of cognitive functions in PwPD: computer-based platforms, exergames, paper-and-pencil programmes, dual-task or treadmill training with action observation therapy, motor imagery, and virtual reality components, interventions targeting precise cognitive domain, tele-rehabilitation, transcranial direct current stimulation, structured cognitive training, and multimodal treatments. Cognitive functions were assessed employing neuropsychological tests, self-report questionnaires, and computerised batteries. **Conclusions**: Overall, the review reported better performances in the experimental groups compared to the control groups, in several cognitive domains. Structured cognitive training emerged as the most effective strategy to enhance cognitive functioning in PwPD. However, further studies are necessary to determine the most appropriate and useful training and to develop interventions that also consider patients’ quality of life.

## 1. Introduction

Parkinson’s disease (PD) is a neurodegenerative disorder, primarily defined by a triad of motor core features, namely: bradykinesia in combination with either rigidity, rest tremors, or both [1]. These clinical manifestations influence balance and gait through a reduced postural control, that provokes a reduction in stride length and speed, and a higher risk of falls, Freezing of Gait (FoG), and alterations of gait spatiotemporal parameters [2]. This symptomatology results from a neuronal loss in the substantia nigra, which causes striatal dopamine deficiency [3].

Worldwide the prevalence of PD is approximately 0.3%, with an incidence rate ranging from 5 to 35 new cases per 100,000 individuals per year [3], with both rates rising with age. On average the onset is at 70 years old and it is almost twice as prevalent in men than in women in most age groups and populations [3].

The diagnosis is based on the clinical presence of the motor triad; diagnostic accuracy depends on disease duration, age, and clinicians’ expertise [1]. It is important to highlight that early in the disease, error rates for a clinical diagnosis even in specialised centres can be as high as 24%. The most common misclassifications are with atypical parkinsonism (i.e., multiple system atrophy-MSA-, progressive supranuclear palsy-PSP- and, less frequently, corticobasal degeneration-CBD-) or with essential tremor, drug-induced parkinsonism, and vascular parkinsonism [3,4]. Misdiagnosing these conditions could lead to ineffective pharmacological treatments, delayed interventions, and poor patient outcomes [3,4]. Certain features can help differentiate these conditions. For example, idiopathic PD typically shows a good initial response to levodopa (the main pharmacological treatment for motor symptoms), whereas MSA is characterised by prominent autonomic dysfunctions and a poor response to levodopa. In terms of cognitive impairments, executive dysfunction and attention deficits are common across PD, PSP, and MSA, while language and visuospatial impairments are more frequently observed in CBD and PD with dementia [5,6]. Thus, accurate differentiation is crucial for providing targeted medications, therapies, and rehabilitation strategies, managing disease progression, and improving quality of life (QoL) for patients and caregivers [3,4,5,6].

Even when considering only idiopathic PD, the symptomatology covers a wide spectrum and could manifest with a predominant resting tremor (tremor-dominant subtype), or with marked akinesia and rigidity (rigid–akinetic or postural instability/gait difficulty subtype) [7]. This differentiation seemingly underlines major pathological variations. Individuals with the tremor-dominant subtype usually have a slower disease progression than patients with the rigid–akinetic subtype [7]. In fact, the severity of the tremor-dominant subtype does not correlate with the dopaminergic deficit in the striatum, nor with the clinical course of PD, in contrast to the rigid–akinetic subtype [7]. Anticholinergic agents, the first developed medical treatment for the symptomatic treatment of PD, tend to be more effective on tremors, than on akinetic–rigid symptoms; while akinesia and rigidity might show better and earlier response to dopamine replacement therapy [7]. Furthermore, whilst both subtypes have been associated with alterations in basal ganglia and motor cortex, critical not mutually exclusive distinctions have been proposed [7,8]. Specifically, rigid–akinetic symptoms have been linked to altered function and anatomy of basal ganglia motor loops, particularly in projections from the putamen to the globus pallidus, thalamus, and ultimately the motor cortex [7,8]. Instead, tremor symptoms have been associated with altered interactions between the cerebellothalamic circuit and the globus pallidus [7,8]. Thus, considering these relevant distinctions between the tremor-dominant and rigid–akinetic subtypes could help clinicians optimise the management and treatment of PD [3,4,5,6,7,8].

The most commonly employed instruments for the assessment of PD staging and symptomatic characterisation are, in order of application, the “Movement Disorders Society (MDS) Modified Unified Parkinson’s Disease Rating Scale Part I-IV (MDS-UPDRS Part I-IV)” [9], the “modified Hoehn and Yahr scale” (mH&Y) [10], the “Activities of Daily Living” (ADL) [11], and the “Instrumental Activities of Daily Living” (IADL) [1,12]. Nonetheless, over the decades, it has become apparent that the impact of a multitude of non-motor, neuropsychiatric, and cognitive symptoms is associated with PD. These symptoms can emerge before the onset of the motor core features [1,3]. Related to cognitive symptoms, widespread intracellular protein (α-synuclein) accumulation and Lewy bodies (aggregations of α-synuclein) are considered the main factors that may lead to the risk of dementia in PD [3].

Non-motor symptoms (NMS) encompass sleep disturbances, bladder disorders, gastrointestinal manifestations, fatigue, autonomic dysfunction, and sensory abnormalities [13]. Dysautonomia, a prominent component of autonomic dysfunction, is increasingly recognised as a significant feature not only in atypical parkinsonism but also in PD, with substantial implications for diagnosis and management [14]. While this study focuses on cognitive symptoms and their rehabilitation in PD, it is important to acknowledge that autonomic dysfunction can contribute to the complexity of non-motor symptomatology in PD [14,15]. Indeed, additional rehabilitation approaches (e.g., such as strategies to manage autonomic dysfunction and to improve daily functioning) are needed to address dysautonomia in PD [1,14,15,16]. Moreover, rehabilitation in PD should be individualised to account for both motor subtypes, and clinical comorbidities, including mood disorders, sleep disorders, osteoporosis and fractures, musculoskeletal pain, and peripheral neuropathy [1,3,16]. For instance, individuals with tremor-dominant PD might benefit more from interventions targeting fine motor skills and tremor reduction; while individuals with postural instability/gait difficulty might require focus on balance, gait, and postural control [2,17,18,19,20,21].

Tailoring rehabilitation to these different aspects and comorbidities of PD is a considerable strategy to optimise functional outcomes and the quality of life [1,3,16].

Even though NMS are widespread in people with PD (PwPD), being the presenting clinical characteristic of PD in over 20% of individuals [16], they are still underdiagnosed and undertreated [13]. Neuropsychiatric comorbidities include disorders of affects (e.g., depression and anxiety), hallucinations and other psychotic experiences, impulse control disorders, anhedonia, and apathy [22,23]. The reported prevalence of these symptoms is around 70 to 89%, with both the frequency and severity increasing over time [23]. Moreover, such symptoms appear to result from a “perfect storm” of contributing factors, including demographic characteristics, diffuse and multiple neurodegenerative disease pathologies, other neurobiological factors, and PD medications themselves [24]. Neuropsychiatric manifestations were found to be associated with cognitive impairment in PD [25], which has been recognised as the most problematic clinical entity among NMS [24]. Besides α-synuclein accumulation and Lewy bodies, the manifestation of cognitive deficiencies may result in an increased risk of developing a mild cognitive impairment associated with PD (PD-MCI), characterised by impairment in one or more cognitive abilities without a negative impact on the autonomies of daily living [26], to a full dementia syndrome associated with PD (PD-D) [27,28]. Precisely, the most frequently impaired cognitive domains in PwPD, that should be assessed by a comprehensive neuropsychological evaluation, are the following: executive functions, attention, working memory, language, long-term memory, episodic memory, visuocognition, and visuospatial abilities [26,28]. Nonetheless, also cognitive deficits in idiopathic PD are highly heterogeneous and often linked to motor signs and disease progression. Late-onset PD without tremors carries a higher risk of dementia, while tremor-dominant patients tend to preserve cognitive function longer, reflecting potential differences in pathology. Cognitive impairments range from dopamine-sensitive fronto-striatal dysfunction affecting executive functions to cortical dementia involving visuospatial and memory deficits, often tied to Lewy body or amyloid pathology [5,6,29,30,31].

Interaction between cognitive and motor functioning in PD might be detected in the mirror neuron system (MNS), which consists of a group of visuomotor, audiovisual, and sensory neurons located in parieto-frontal and limbic systems. These neurons are activated when performing an action, through vision, hearing, and proprioception, and while imagining to execute a movement [2]. Therefore, MNS is implied in motor planning, motor learning, or relearning cognitive processes [2]. Coherently, the literature has identified action observation therapy (AOT) and motor imagery (MI) as two of the most appropriate strategies based on the MNS for motor relearning in PwPD [2,18,32]. Moreover, the literature outlined improvements for individuals with PD experiencing FoG who underwent rehabilitation with AOT or MI components [2,18].

Noteworthy, findings highlighted a strengthening in language, executive functions, attention, memory, verbal memory, visuospatial memory, and working memory as a result of these interventions, in particular when employed within a dual-task framework [2,18]. This evidence is probably due to the potential modulation of the brain plasticity activated by these mental practice techniques [2,18].

### Objectives

The clinical presentation of PwPD is exacerbated by the presence of the above-mentioned non-motor, neuropsychiatric, and cognitive manifestations, afflicting subjects and caregivers’ disease burden and QoL, with higher rates of institutionalisation [22,28]. Over time, available valid treatments for motor manifestations in PD have increased [17]. Conversely, programmes for the rehabilitation of cognitive symptoms remain limited; an issue that is even more relevant if considering the several comorbidities (e.g., mood disorders, sleep disorder, autonomic dysfunction, and peripheral neuropathy) [1,3,14,15,16] and PD subtypes, i.e., tremor-dominant subtype and rigid–akinetic subtype [17,19].

The rigid–akinetic subtype, characterised by postural instability and gait difficulties shows more cognitive impairment and significantly worsens with increasing disease severity compared with patients with the tremor dominant phenotype [19].

Currently, there is no proven disease-modifying pharmacotherapy for PD. Available treatments, including dopaminergic medications (e.g., levodopa) aim to alleviate symptoms and help restore dopamine levels in the brain, rather than alter the underlying disease progression [3]. Despite pharmacological therapy is considered the goal standard for the treatment of both motor and non-motor symptomatology in PD; potential adverse effects, comorbidities, and the evolution of motor symptoms (e.g., drug-induced dyskinesia) complicate the effects of drug treatments [3,17,22,33]. In particular, pharmacological treatments can impact both cognitive function and rehabilitation outcomes [22,26,28]. Levodopa might have various effects on cognition, improving some cognitive functions but potentially causing cognitive fluctuations or even worsening executive function [3,17,22,26,28,33]. Additionally, medications used to manage NMS, such as dopamine agonists or antidepressants, could also influence cognitive processes [22,26,28].

Furthermore, PD might overlap with other neurodegenerative conditions, such as Alzheimer’s disease (AD), with cortical amyloid-beta and tau pathologies sometimes co-occurring. These interactions may exacerbate cognitive decline and highlight the complexity of managing cognitive training in PD populations [5,31,34]. Non-dopaminergic systems, including noradrenergic and cholinergic pathways, contribute to cognitive and neuropsychiatric symptoms, further complicating therapeutic interventions. These overlaps and comorbidities highlight the importance of tailored cognitive training approaches that consider both the multifactorial nature of cognitive decline and the individual’s cognitive and pharmacological profile to optimise rehabilitation outcomes.

Cognitive rehabilitation follows a restorative or compensatory approach, improving cognitive functions, or employing training strategies to accommodate cognitive impairment, especially in the later phases of the disease [17]. Through experiential or environmental stimulation, cognitive interventions enhance neural networks of attentional and control process via neuroplasticity, while employing feasible and low-cost rehabilitative strategies (e.g., paper-and-pencil exercises) [17]. Moreover, cognitive training might increase grey matter volume, and frontal lobe function by activating mechanisms of brain plasticity, and memory-related hippocampal function [17]. Even though, several ameliorations were found for various cognitive domains in PwPD, the transferability of these improvements to other clinical and non-clinical aspects (such as ADL, balance, FoG, etc.) is still limited [17].

Consequently, research should focus on developing suitable and effective interventions targeting not only the rehabilitation of the core motor features of PD but also the numerous cognitive and non-motor symptoms (NMS).

From the first endeavours on the theme [35,36], several reviews have discussed this issue. Some articles emphasised treatments involving embodied cognition, MI, AOT, and mirror neurons in PD [2] or in other neurodegenerative disorders [18]. Differently, Lawrence and colleagues [37] did not include these interventions but encompassed studies employing non-invasive cognitive training as well as transcranial direct current stimulation (tDCS). Otherwise, different reviews did not comprise studies involving these types of treatment and, moreover, did not specifically include randomised controlled trials [38,39]. In 2020 a Cochrane database review addressed the topic, but focused solely on PD-D and PD-MCI populations [17].

Therefore, the present systematic review aimed to ascertain randomised controlled trials (comparative, prospective studies performed under controlled conditions with random participants allocation) or randomised clinical trials (concerning a comparison between two different treatments) regarding precise cognitive training targeting an enhancement for cognitive functions in PwPD. Also, this systematic review considered cognitive training targeting other motor and NMS and did not focus on a specific sub-population (such as solely individuals with PD-MCI). The review selected interventional studies concerning cognitive training, encompassing the use of traditional or technological tools, tDCS, multi-domain training programmes, AOT and, MI components. To the best of our knowledge, the present review is the first attempt to compare cognitive training in PD.

## 2. Materials and Methods

The present review followed the “Preferred Reporting Items for Systematic Reviews and Meta-Analyses” (PRISMA) guidelines [40] and a completed PRISMA checklist is available in the Supplementary Materials (File S1). The review protocol was registered in the online International Prospective Register of Systematic Reviews (PROSPERO) (# CRD42024570141). The online platform “Rayyan” [41] was employed for the de-duplication and screening of the papers.

### 2.1. Search Strategies

Following the PRISMA guidelines [40], systematic searches of the papers were conducted from January 2024 to May 2024. The databases considered were “PsycINFO”, “PubMed”, and “Web of Science”, for the retrieval of articles from inception to the end of January 2024. Detailed research strings entered in the databases are presented in Table 1.

### 2.2. Inclusion and Exclusion Criteria

English, peer-reviewed, full-text, human studies concerning cognitive training, structured as randomised controlled trials or randomised clinical trials in PwPD, were considered for the review. Focusing on the training of cognitive functions, we included programmes concerning: the use of technological tools (i.e., exergames, virtual reality), action observation therapy, motor imagery components, traditional methods for cognitive training, executive functions training, multidomain programmes, dual-task interventions, and cognitive training targeting other motor and non-motor symptoms of PD.

Animal studies, articles considering clinical populations other than PD (parkinsonism), papers not written in English, not peer-reviewed research, pilot studies, feasibility and/or sustainability studies, studies without a cognitive outcome, single session/no-training studies, and any other type of study design besides randomised controlled trials or randomised clinical trial were excluded.

### 2.3. Screening and Study Selection

The results obtained by entering the research string in the considered databases were screened employing the online platform “Rayyan” [41]. Following the PRISMA guidelines [40], after the de-duplication process, articles were evaluated by title and abstract by two independent reviewers (MFG; SG), in order to identify potentially includible papers, excluding those that did not meet the eligibility criteria. A third reviewer (FLS) resolved eventual inconsistencies. Then, the full texts were independently retrieved and assessed for eligibility following inclusion/exclusion criteria by the review team (MFG; SG). The third researcher (FLS) was consulted to solve doubts/conflicts.

### 2.4. Data Extraction and Analysis

Data extraction was performed by the same two independent reviewers (MFG; SG) and confirmed by the third one (FLS). Information gathered from each article included general details (i.e., author, year of publication, title, journal, country, number of participants, study design), patient information (including diagnostic criteria, age, gender, comorbidity, staging, and PD symptomatic characterisation), experimental and control treatments, targeted cognitive functions (e.g., cognition, motor imagery, executive functions, attention, memory), and measures employed to assess them (self-report, cognitive test, neuropsychological scales). Furthermore, mean scores and standardised deviations of variables of interest, performed statistical analyses, information about the reliability and validity of outcome measures, ethical approval, and standardised protocols were retrieved from each included article.

Given the variety of trial designs and the heterogeneity of the data, no sub-analysis or meta-analysis was planned or performed. Consequently, the final form of data extracted and reported in the present review was discussed for purely descriptive purposes.

### 2.5. Quality Assessment of Included Studies

Concerning the risk of bias assessment, two independent reviewers (MFG; SG) performed the evaluation, and a third reviewer (FLS) intervened in case of discrepancies. Since the present systematic review included only randomised controlled trials or randomised clinical trials, the “Revised Cochrane risk-of-bias tool for randomised trials” (RoB 2) was employed [42]. RoB 2 is a validated and recommended instrument composed of 5 assessed domains: “risk of bias arising from the randomisation process”, “risk of bias due to deviations from the intended interventions”, “risk of bias due to missing outcome data”, “risk of bias in the measurement of the outcome”, and “risk of bias in the selection of the reported result”.

## 3. Results

### 3.1. Search Result

**Figure 1:**
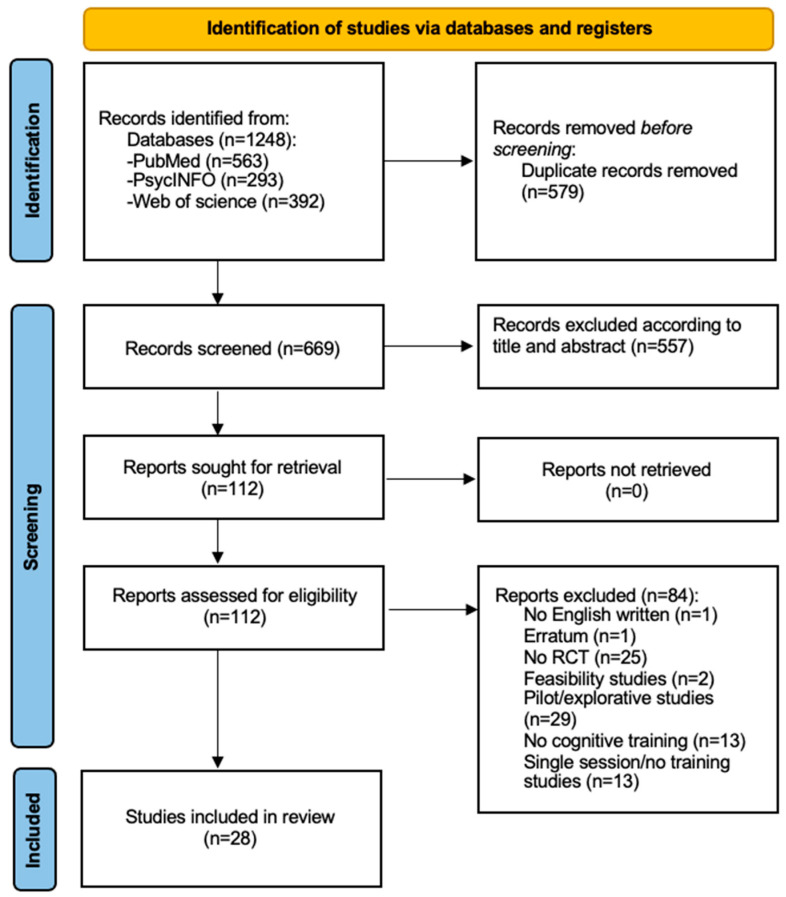
PRISMA Flow Diagram.

The research string entered in the databases led to the obtainment of 1248 results: 563 from “PubMed”, 293 from “PsycINFO”, and 392 from “Web of Science”. The 1248 results were subsequently de-duplicated, reaching a total of 669 potentially relevant articles submitted to the first independent screening per title and abstract. At the end of the first evaluation, 112 studies were retrieved for the independent full-text analysis. Eighty-four papers were excluded, mostly due to the design of the study, either being pilot research (n = 29) or not being a randomised controlled trial (n = 25); thus, leading to the final inclusion of 28 studies in the present review. Figure 1 illustrates the PRISMA flow diagram for each stage of the systematic review.

### 3.2. Study and Sample Characteristics

The 28 studies included in the review were conducted in 10 different countries, precisely 8 in Italy [43,44,45,46,47,48,49,50], 5 in Germany [51,52,53,54,55], 3 in the Netherlands [56,57,58], 4 in Brazil [59,60,61,62], 2 in the United States of America [63,64], 1 in Colombia [65], 2 in Spain [66,67], 1 in Switzerland [68], 1 in Israel [69], and 1 in Australia [70]. The included trials were performed over a period of time between 2006 and 2024.

Mainly, the studies enrolled PwPD from inpatient (hospital, rehabilitation centre, or clinic) or outpatient (telerehabilitation, telemedicine, ambulatory) settings.

The majority of the studies were structured as randomised controlled trials (n = 19) [43,44,47,48,51,52,53,54,56,57,58,59,63,65,66,67,68,69,70]; only eight studies were randomised clinical trials [45,46,49,50,55,60,62,64], and one study comprised crossover features in its design [61] (further information is reported in Supplementary Materials, Table S1).

Concerning the characteristics of the samples enrolled in the 28 articles, 15 articles reported focus on individuals with mild cognitive impairment associated with PD (PD-MCI) [43,44,45,47,48,49,51,54,55,56,58,59,63,67,70], while the remaining 13 studies did not report this inclusion criterion [46,50,52,53,57,60,61,62,64,65,66,68,69]. In addition, two trials included individuals with the diagnosis of PD Postural Instability and Gait Disorder (PIGD) phenotype [45,49], and one study highlighted the occurrence of FoG in the sample [50].

In total 1557 subjects participated in the distinct studies. On average, the participants were mainly males (n/males: 881 (56.58%) vs. n/females: 676 (43.42%)). The mean age was 66.70 (S.D. 3.52), ranging from 50 to 80 years. Regarding PD staging and symptomatic characterisation, overall, the mean score of UPDRS-III was 27.01 (S.D. 6.96), while the mH&Y scale ranged from 1 to 4.

The general information about the studies, including the characteristics of the samples, the trials’ exclusion and inclusion criteria, and the country of the studies, is reported in the Supplementary Materials (Table S2).

### 3.3. Quality Appraisal

**Figure 2:**
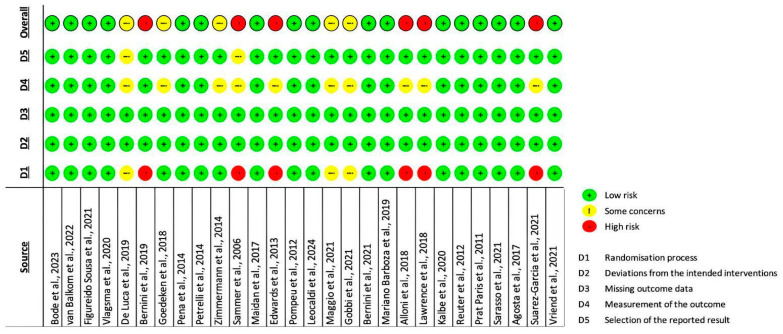
RoB 2 assessment results [43,44,45,46,47,48,49,50,51,52,53,54,55,56,57,58,59,60,61,62,63,64,65,66,67,68,69,70].

The risk of bias assessment conducted employing the RoB 2 tool [42] outlined the preponderance of the included articles (n = 17) as high-quality studies, indicating a low risk of bias [45,47,49,50,51,52,54,55,56,57,58,59,60,62,66,67,69]. Six articles [44,48,53,64,65,70] resulted in a “high risk” of bias, mostly due to issues related to the first domain (i.e., “risk of bias arising from the randomisation process”); while five studies were evaluated as having “some concerns” [43,46,61,63,68], generally because of controversies concerning the measurement of the outcome domain. Nevertheless, no studies were excluded from the present review because of the quality appraisal. The detailed results of the risk of bias assessment are described in Figure 2.

### 3.4. Data Extraction

Given the variety and heterogeneity of the training programmes, and the different methods used to assess cognitive functions, the inherent data gathered from the articles were divided into three tables reporting, respectively, cognitive assessment, rehabilitative interventions, and the effects of the latter on cognitive functioning.

Table 2 delineates the principal information regarding each included trial, considering their study design and main results. Moreover, a table summary with all the further information retrieved from each research is provided in Supplementary Materials (Table S1).

#### 3.4.1. Assessment of Cognitive Functions

Referring to the evaluation of cognitive functioning, the preponderance of the included studies examined cognition and/or distinct cognitive functions as their primary outcome [43,44,45,46,47,48,49,52,53,54,55,56,57,59,61,62,63,64,65,66,67,68,69,70], and only four studies as their secondary outcome [50,51,58,60].

The assessment was conducted employing, in order of frequency use, neuropsychological standardised and validated paper-and-pencil tests [43,44,46,47,48,50,51,52,53,54,55,58,59,60,61,62,66,67,68], a computerised battery of cognitive evaluations [45,49,69,70], self-report questionnaire filled autonomously by the patients [57,58,63], and the performance of designated virtual tasks [56,57,64,65]. Therefore, the evaluation could be performed either directly by a trained neuropsychologist, registered by automated platforms or according to the participant’s reported subjective complaint. In addition, some studies included neuroimaging techniques to detect changes in cerebral regions in PwPD after the different rehabilitation programmes [45,49,50,56,69]. Whilst, some articles reported to have carried out a long-term follow-up evaluation after the end of the trial programmes [44,48,54,55,57,62].

Considering outcomes, seven studies [45,46,50,59,61,62,69] did not distinguish cognition in separated domains, compared to the majority of the included articles that differentiated the cognitive outcomes [43,44,47,48,49,51,52,53,54,55,56,57,58,60,63,64,65,66,67,68,70]. In detail, cognitive functions were usually categorised into global cognition, attention, visuocognition, visuoconstruction, language, logical-executive functioning, frontal abilities, memory, working memory, verbal memory, visual memory, and processing speed.

As stated before, overall, the performed cognitive assessment comprised a wide variety of cognitive measurements. Focusing on neuropsychological assessment tools, the “Montreal Cognitive Assessment” (MoCA) [71], the “Mini-Mental State Examination” (MMSE) [72], the “Addenbrooke Cognitive Examination-III” (ACE-III) [73], and its revised form (ACE-R) [74] were the most applied for the assessment of global cognition. Concerning language, the “Aphasia Check List” (ACL) [75] and the “Boston Naming Test” (BNT) [76] were administered to PD patients. For the attentive domain, the “Trail Making Test” (TMT) [77], the “Attentive Matrices” [78], and the “Stroop Colour and Word Test” (SCWT) [79], were often utilised. The TMT and SCWT tests were used for the evaluation of processing speed and executive functions, in the latter case, in association with the “Weigl’s Sorting Test” (WEIGL) [80], the “Frontal Assessment Battery” (FAB) [81], the “phonemic and semantic word fluency test” [82], the “Battery of Behavioural Assessment of the Dysexecutive Syndrome” (BADS) [83], the “Raven’s Coloured Progressive Matrices” (RCPM) [84], and the “Tower of London task” (ToL) [85]. Regarding verbal memory, the “Rey Auditory Verbal Learning Test” (RAVLT) [86] and the “California Verbal Learning Test-II” (CVLT-II) [87] were performed in most of the encompassed studies. The “Corsi’s block-tapping test” (CBTT) [88], the “Wechsler Adult Intelligence Scale” (WAIS) [89], and the “Digit span” [90] were mainly used for working memory. The “Clock Drawing Test” (CDT) [91], the “Benton judgement of line orientation” [92], and the immediate recall of the “Rey-Osterrieth Complex Figure Test” (ROCFT-ir) [93] were carried out to assess visuocognition and visuoconstruction; instead, the ROCFT delayed recall (ROCFT-dr) was the most used instrument for visual memory.

Lastly, it is worth mentioning that the majority of the considered papers reported other outcomes related to clinical, psychological, and motor functioning, due to their strict relationship with cognition in PD [43,46,49,50,51,52,53,54,59,60,61,62,64,65,66,67,68,69,70]. Furthermore, some studies also considered the caregivers’ emotional state. Further detailed information is available in Supplementary Materials (Table S1).

#### 3.4.2. Cognitive Training

Considering the duration of cognitive interventions, they ranged between a few days of training [63,64,65] and 32 weeks [61], with an overall duration of 4–6 weeks (M = 6.61, S.D. = 6.05) of rehabilitation. Regarding the intensity of the treatments, participants performed around one to four exercise sessions per week.

The considered studies applied various experimental interventions for the training of cognitive functions in PwPD; among which were identified: computer-based cognitive platforms, exergames, paper-and-pencil cognitive programmes, dual-task or treadmill training (TT) with AOT, MI, and virtual reality (VR) components, interventions targeting a precise cognitive domain (i.e., executive functions, processing speed, prospective memory), tele-rehabilitation programmes, tDCS, structured cognitive training, and multimodal treatments. The preponderance of the trials employed computer-based cognitive programmes either for practising different cognitive domains or as a tool for specific interventions [43,44,46,47,48,55,56,57,58,60,63,64,67,68,70]. The most used platforms were: “SmartBrain Pro” (http://www.smartbrain.net), “Cogniplus” [94], “CoRe software” [95], and “Braingymmer” (www.braingymmer.com; Dezzel Media). One study [67] utilised a combination of computer-assisted and paper-and-pencil cognitive training, while another one [59] included only printed materials. A single paper [60] performed Nintendo WiiTM-based exergames (Nintendo, Redmond, WA, USA) as experimental cognitive–motor intervention; instead, as will be discussed below, another research [68] allocated participants to Nintendo WiiTM exergames as a control condition. Among computer-assisted training, three trials developed tele-rehabilitation or home-delivered programmes: one for general cognitive exercise [56], one encompassing components for cognitive and socio-cognitive stimulation [46], and one specific for the exercise of processing speed [64]. Moreover, another single-domain intervention, focusing on prospective memory, included a computer-based “Virtual Week” platform [63,96]. The remaining targeted treatments included executive functions training, either with or without the implementation of computerised tools [48,53,58]. Referring to structured cognitive training, three studies [51,52,54] employed the “NEUROvitalis Parkinson training” [97], while one research [66] utilised the “cognitive rehabilitation programme in psychosis (REHACOP)” [98]. Four articles [44,55,61,62] created multimodal interventions, including motor, balance, cognitive, psychological, social, occupational, functional, and transfer exercises, either with computer-assisted or paper-and-pencil tasks; even considering a “switch” between the several activities [61]. In relation to tDCS, two studies included stimulation in their procedure, one in addition to other computerised training conditions [70], another as the solely experimental treatment [65]. Finally, four trials integrated treadmill or dual-task training with AOT, MI, or VR components [45,49,50,69].

Regarding control conditions (which were the different comparison treatments in randomised clinical trials) the encompassed studies involved: waiting list [52,64], physical practice [44,51,54,62,69], paper-and-pencil [43,47], or computerised cognitive programmes [56,58], exergames [68], sham interventions [48,65], active control groups [46,57,60,66,67], standard treatments [53,59], and tailored restrained conditions [45,49,50,55,61,63,70]. On average, the most employed “standard” control condition was the active control group. To consult additional details, Supplementary Materials (Table S1) provides an exhaustive summary table.

#### 3.4.3. Effect of Cognitive Training on Cognitive Functioning

Almost the totality of the considered studies reported, to some extent, a cognitive enhancement in their participants. Overall, the studies recurrently reported better performances in the experimental groups compared to the control groups in global cognition [44,48,50], attention [45,46,47,51,59,61,67,68,70], processing speed [47,59,64,66,67], executive functions [43,45,46,51,52,53,54,59,61,67,70], visuospatial abilities [43,46,62,67], memory [48,61,66,67,70], short-term memory [52,62], and working memory [52,61,70]. Other improvements that were not often addressed comprised either cognitive functions (i.e., visuoconstruction, visual memory, verbal fluency, attention shifting) or psychological and motor aspects (e.g., QoL, stress, ADL, FoG, fall rates).

Examining in depth each category of cognitive interventions, computerised multi-domain training described improved performances in global cognition, visuospatial and executive functions, when compared to paper-and-pencil cognitive rehabilitation [43], in one case with medium/large effect sizes [47].

Participants enrolled in an experimental programme with the use of Nintendo Wii exergames obtained a higher enhancement in global cognition, in ADL, and in balance, against PwPD in the physical exercise treatment [60]. On the same topic, in another trial [68], individuals who received the Wii-based exergames intervention showed better improvements in attention with a small effect size, compared to the “Cogniplus” training condition [94].

A study based on the “Braingymmer” (www.braingymmer.com; Dezzel Media) online platform reported the absence of effect on their primary and secondary outcomes after treatment [57]. Similarly, another study employing the same software in a home setting only reported an improvement in ToL response time [56]. Remaining on the tele-rehabilitation/home-delivered interventions, Maggio and colleagues [46] reported cognitive, social, and psychological improvements in the Tele-VR group with respect to the paper-and-pencil control group. Regarding single-domain training, computer-assisted home-based intervention resulted in improvements in processing speed with a small effect size [64]. In a different setting, a computerised programme specific for prospective memory outlined a decline in the control condition, rather than improvement in the experimental group [63]. Instead, executive functioning computer-based training resulted in success and even led to better global cognition and memory [48]. Always referring to this cognitive domain, standard not-computerised intervention [53], presented enhancement only in a few numbers of BADS’s subtests [83]; while the “ReSET—Strategic Executive Treatment” (ReSET) did not arouse executive functions [58].

Focusing on Sousa and colleagues’ paper-and-pencil cognitive training [59], compared to the standard general intervention performed in their rehabilitative setting (various activities, including physiotherapy, speech therapy, physical activity, etc.), this treatment was beneficial for strengthening processing speed, shifting attention, verbal fluency, and global cognition An integration of the last-mentioned programme with computer-assisted tasks reported large and medium effect sizes for ameliorations in attention, information processing speed, memory, visuospatial, and visuoconstructive abilities, semantic verbal fluency, and executive functions [67].

In relation to tDCS, its application as the solely experimental condition was effective for enhancing action-verb processing with medium/large effect sizes [65]; whilst the addition of tDCS to computerised standard and tailored cognitive training improved performances in attention, working memory, memory, and executive functions [70].

A multimodal treatment developed by Bernini and colleagues [30] structured with an integration of computer-aided cognitive exercises and standard motor exercises was successful in increasing participants’ global cognition and executive functions compared to only the physical activity control group, reporting medium/large effect sizes [44]. Another research was designed in a similar manner, combining balance-motor training with cognitive practice, but in a paper-and-pencil format, and conducted to improvements in short-term memory and visuospatial abilities either for experimental or control conditions [62]. Reuter and colleagues developed a cognitive, transfer, and psychomotor programme with continuation at home, that led to improvements in both primary and secondary cognitive outcomes [55]. Their intervention was characterised by cognitive and motor exercises, which had to be transferred to their daily life activities in order to improve their quality of life. Lastly, a multimodal training with cross-over features reflected an improvement in attention and working memory with medium effect sizes [61].

Analysing structured cognitive training, three distinct studies employed the “NEUROvitalis Parkinson training” [97], comparing the treatment, respectively, to physical activity [51,54], unstructured rehabilitation and waiting list control groups [52]. All these articles outlined cognitive enhancement, precisely in working and short-term memory (medium to large effect sizes) [52], and in executive functioning [51], especially in verbal fluency (medium effect size) [54]. Furthermore, another structured intervention, the “REHACOP” programme [98], resulted in significant differences between experimental and occupational therapy as control conditions in processing speed, theory of mind (TOM), visual learning and memory, with moderate and large effect sizes [66].

Ultimately, TT or dual-task training in addition to AOT, MI, or VR components resulted in an improvement both in motor outcomes (e.g., balance, FoG, speed, mobility) measured with “Timed-Up-and–Go” and “Timed-Up-and–Go-Cognitive” tests (TUG, TUG-COG), QoL, and cognitive functions with increased attentive–executive domains and different patterns of brain activation during imagined obstacle negotiation [45,49,50,69].

Supplementary Materials (Table S1) highlight further information on the effects of cognitive training.

## 4. Discussion

Over time, scientific literature has underlined the efficacy of non-pharmacological rehabilitative approaches targeting the classical motor symptoms, balance, gait speed, freezing, and QoL in addition to pharmacological treatment in PwPD [3,22,33,99]. Indeed, even if pharmacological therapy is considered the goal standard for the treatment of both motor and non-motor symptomatology in PD [3,22,33], the administration of levodopa supplementation and psychiatric medications is complicated by potential adverse effects, the presence of comorbidities, and the evolution of motor symptoms, including drug-induced dyskinesia [3,17,22,33]. Conversely, non-pharmacological interventions incorporate physiotherapy, physical exercise-based strategies, tDCS, yoga, and Tai Chi training [2,3,22,33,99]. During the last decades, a growing interest in non-pharmacological rehabilitation for the cognitive and neuropsychiatric symptoms of PD emerged in clinical research, favoured by the recognition of cognitive impairment as the most problematic clinical entity among NMS [1,22,24,26]. Several treatments resulted in effective: mindfulness, cognitive training, cognitive-behavioural psychotherapy, multidomain therapy, exergames, and computerised rehabilitative platforms [22,38,39,99]. However, structured studies about non-pharmacological therapies with robust methodologies and medium to long-term effect evaluations are needed to address the clinical complexity of PD.

Therefore, the current systematic review aimed to investigate available cognitive training of PwPD, including trials targeting other motor and NMS. The review selected interventional studies concerning cognitive training encompassing the use of traditional or technological tools, tDCS, multidomain training, AOT, and MI components.

Twenty-eight studies were included in the present review, focusing on cognitive training in PD. Concerning the characteristics of the enrolled participants, evidence was coherent with the literature stating a higher prevalence of PD in males and an average onset at 70 years old [3]; while mild to moderate UPDRS-III levels reflected the importance of early intervention to reduce the risk of exacerbations [99,100,101].

In general, studies were heterogeneous in relation to the design of cognitive assessment and intervention, thus no sub-analysis or meta-analysis was performed. Regarding the evaluation of cognitive functioning, the majority of the papers conducted an extended neuropsychological assessment distinguishing the various cognitive domains. This evidence was coherent with the literature, outlying the importance of an extensive and in-depth neurocognitive assessment conducted by an expert, in, at least, the following domains: global cognition, attention, working memory, executive functions, language, visuospatial cognition, and episodic memory [22,26,28]. Moreover, the administered neuropsychological tests were consistent with clinical recommendations for PwPD [26,28]. This is a crucial aspect to consider since cognitive impairment is often associated with other neuropsychiatric manifestations; thus, contributing, in combination with demographic characteristics, other neurobiological factors, and PD medications themselves, to patients’ and caregivers’ disease burden and invalidated QoL [22,23,24,25,26,28]. Furthermore, a complete neuropsychological evaluation provides a better outcome detection compared to a self-report questionnaire and/or subjective performance at computerised platforms, which might be invalidated by methodological bias and that does not clearly distinguish the several cognitive domains [22,26,28].

Examining the various categories of cognitive training implemented by the considered studies, results were coherent with findings from previous systematic reviews, also referring to a frequent application of computerised experimental tools and active control conditions [2,38,39,101]. Although the notable heterogeneity in training design, overall, the studies reported better performances in the experimental groups compared to the control groups, in global cognition, attention, processing speed, executive functions, visuospatial abilities, short-term memory, and working memory. These improvements occurred regardless of the treatment duration, intensity, and structure, with recurrently reported medium to large effect sizes. Consequently, it might be affirmed that the cognitive training “per se” is conducted for overall improvements and that it might be crucial to intervene in cognitive symptoms, regardless of the chosen treatment. However, publication bias must also be considered while making this assumption, since until some years ago it was difficult to publish a clinical trial without relevant findings; even though these null results are extremely helpful in orienting future research. Therefore, a reflection on the risk of bias in the included studies must be articulated.

In the present systematic review, no trials were excluded because of the quality appraisal, which highlighted a low risk of bias for the preponderance of the included articles. Nonetheless, for a purely descriptive purpose, it is important to distinguish the results obtained by studies with a robust methodology from the ones derived from trials with methodological pitfalls. Precisely, among trials emerged with a low risk of bias, structured cognitive programmes [51,52,57,66] and dual-task/TT with AOT-MI, or VR components [45,49,50,69] were the most effective in enhancing cognitive functioning, with medium to large effect sizes. On the other hand, as will be further investigated below, other studies with the same result at the quality appraisal did not describe cognitive improvements [56,57,58], probably due to an issue related to the performed measurements.

Instead, an article that resulted in “some concerns” in the domains related to outcome detection, effectively did not refer to ameliorations [63]. The finding might be connected to these methodological issues. Similarly, tele-rehabilitation or home-delivered interventions resulted in some concerns. Given their unsupervised structure (due to training being performed at home without assistance by PwPD), their improvements should be interpreted cautiously [46,64]. Moreover, studies with the addition of various components or with cross-over features [61,70] should be carefully considered. The emerging problems in their quality appraisal might be related to a “heavy design”, with several distinct training groups; therefore, a tailored structure might be more suitable. Interestingly, in the 2019 study by Bernini and colleagues [44], a high risk of bias was obtained, especially due to the absence of randomization, and an inappropriate comparison condition (standard physical training), while referring improvements with medium to large effect sizes; in their subsequent trial of 2021 [47], these methodological pitfalls were not observed. This last article [47] emerged with a low risk of bias, and given the similar type of cognitive training (both being computerised), it should be taken into greater consideration than its predecessor.

Analysing in depth each structural characteristic of the studies, the average duration was around 4/6 weeks of rehabilitation with one to four exercise sessions per week. This assumption was consistent with the literature suggesting a high-intensity intervention for better outcomes in PwPD [22,99,102]. Accordingly, some of the included articles, that did not report significant results, were structured with a low-intensity and/or duration design [58,63,64]. Another possible explanation pertains to the outcome detection of these studies, coherently with the above-mentioned assumptions on the risk of bias assessment; since a self-paced version of neuropsychological tests [57,64], or self-referred measurements was employed [63]. In addition, researchers indicated neuropsychological assessment performed by an expert as the goal standard evaluation [22,26,28]. A further related critical aspect was the absence in most of the encompassed studies of a long-term follow-up, considered relevant for detecting progress maintenance by scientific consensus [38] and even highlighted as a limit by some of the studies [43,44,64,66,67].

As stated before, in general, the considered treatments resulted in various cognitive improvements. However, it is essential to examine the structure of the applied control or comparison conditions. In fact, various trials described improved performances in cognitive training groups while weighed against a different category of treatment (e.g., physical training, sham intervention, unstructured activities), or even against a solely waiting list control condition [64]; thus, making a proper effect comparison difficult [38]. Furthermore, only two articles considered the addition of a control group of PwPD who did not undergo training [52,70]; whilst other studies encompassed additional elements such as psychoeducation, transfer training, or unspecific aspects, therefore complicating the determination of the influences on the results.

Overall, structured cognitive training (i.e., NEUROvitalis, REHACOP) appeared as the most effective category of cognitive interventions, since all these programmes outlined enhancements in their participants, precisely in working memory, executive functioning, short-term memory, verbal fluency, processing speed, TOM, visual learning and memory, with moderate to large effect sizes [51,52,54,66]. These findings were coherent with literature reporting cognitive improvements related to structured interventions [22,26,38] and should be wisely considered in future studies since these trials employed a robust methodology. Given the widespread use of computerised platforms in the included papers, it is worth mentioning their obtained improvements in global cognition and executive functions [43,44,47]; evidence in line with assumptions from other reviews [17,38]. Noteworthy, the employment of Wii-based exergames led to better performances in participants either as experimental or control conditions when compared to physical training or computerised cognitive programmes [60,68]; constituting an interestingly economic tool to provide cognitive training. An additional affordable strategy to enhance cognition in PwPD might be individuated in tele-rehabilitation/home-delivered treatments. In the present review, these study designs succeeded in strengthening not only single-domain cognition, like processing speed [64], but also social and psychological functioning [46]; however, careful reflections should be made, due to their issue related to the quality appraisal. Remaining on the issue of single-domain interventions, interestingly, treatments targeting uniquely executive functioning reported minimal or no improvements for training with this cognitive domain as the primary outcome [53,58]; instead, more effects were reported in cases where a different primary outcome was used, such as global cognition [48]. The topic is very crucial and needs to be properly ascertained since executive functions are often impaired in individuals with PD, are associated with other neuropsychiatric manifestations, and have a pivotal role in patients’ everyday lives, ADL, and IADL [22,23,24,25,26,28]. Related to this concern, a category of non-pharmacological interventions that had been discussed in the literature for their potential efficacy on both neuropsychological and psychological functioning is tDCS [3,17,22]. In particular, evidence reported useful tDCS programmes for depressive symptoms in PD [22], which is one of the most occurring neuropsychiatric comorbidities in this neurodegenerative disorder [23,24]. Regrettably, the articles employing tDCS included in the current systematic review did not consider psychological functioning; nonetheless, regarding cognition, enhancements were found for action-verb processing, attention, memory, working memory, and executive functions [65,70]. Lastly, another impaired important aspect of PD is the interaction between cognitive and motor functioning [2,18,35], which is also connected to MNS. Some studies included in the present review [45,49,50,69] might point out this issue, especially, referring to motor planning, motor learning or relearning cognitive processes. In fact, improvements in motor outcomes (e.g., balance, FoG, speed, mobility), TUG and TUG-COG tests, QoL, and cognitive functions were individuated in TT or dual-task training in addition to AOT, MI, and/or VR components [45,49,50,69]. This kind of intervention is not only considered very effective for enhancing MNS-based cognitive functioning [2,18,35], but also, in the present review was implemented with a low risk of bias by all of these trials [45,49,50,69]. Moreover, dual-task training might have the highest ecological validity, mimicking the real-world demands that PwPD are asked to face in their daily living. Studies that used VR [69] for immersive and contextually relevant exercises, integrated real-world tasks [46,55,61] and personalised interventions [55,58] might be more transferable, engaging, and might have higher practical relevance. Monitoring functional outcomes, ADL, and QoL [46,50,53,54,59,60,61,62,67,70], further ensures the relevance and applicability of rehabilitation efforts. Despite being an important issue, none of the included studies discussed the ecological validity of the proposed training.

## 5. Conclusions

Cognitive impairment in PD has been recognised as the most relevant clinical entity among NMS [24], with an increased risk of developing a PD-MCI condition [26], to a full PD-D syndrome [27,28]. In addition, the other non-motor and neuropsychiatric manifestations could exacerbate the clinical presentation of PwPD, compromising their QoL, and increasing the rates of institutionalisation [22,28].

Consequently, the present systematic review aimed to determine the strategies available for the cognitive training of individuals with PD, including cognitive training targeting other motor and NMS. Overall, high-intensity treatment conditions were frequently applied among the included studies, but several distinct categories of cognitive intervention might be beneficial in improving various domains of cognition in PD patients. Among them, coherently with scientific consensus [22,26,38], structured cognitive programmes emerged as the most effective strategy to enhance cognitive functioning in patients with PD [51,52,54,66]. Noteworthy, computerised platforms [43,44,47] and especially exergames [60,68] led to cognitive improvements, constituting an affordable and effective training structure [17,38]. Furthermore, dual-task or TT with AOT, MI, and/or VR components [45,49,50,69] resulted in better QoL, motor and cognitive performances, representing a valid strategy for improving MNS-based cognitive functioning [2,18,32]. However, information regarding the employed methodology and the results from the risk of bias assessment should be wisely considered while examining these trials.

Conversely, contrasting results emerged from the included papers targeting executive functioning [48,53,56,57,58,63,64]. Therefore, further studies with a robust methodology and updated systematic reviews with a specific focus on executive functions are needed to clarify these results. Moreover, considering that our searched terms included the word “training” as bonded to each of the cognitive functions we investigated this might have limited our results. Other limitations of the comprised articles might be attributable to the relatively small samples employed, the absence of long-term follow-up data and a further control group of participants with PD who did not undergo training [38,43,44,64,66,67]. Moreover, some trials inserted in their design additional elements (e.g., psychoeducation, transfer training, unspecific general rehabilitation aspects), thus complicating the determination of the influences on the results. Lastly, the majority of the discussed studies applied training, computerised software, or tools, that are not free of charge. Several considered interventions did not extensively report the employed materials, only referring to other studies on the theme, while various included papers developed their own tailored training. These limitations prevent the reproducibility of the cognitive programmes in future research.

### Future Directions

Further studies are necessary to determine the most appropriate and useful configuration for cognitive training in PwPD. Also, further trials should address neuropsychiatric manifestations, QoL and ecological validity of the proposed training. In general, robust methodologies, long-term follow-up, no-training control group, and a clear outcome detection should be integrated into future research. The literature should focus on the implementation of structured high-intensity open-access interventions, involving a full neuropsychological assessment conducted by an expert, that might be replicated by other researchers. In addition, studies should highlight which techniques are more suitable to improve cognitive functions, considering the possible use of exergames or computerised platforms, given their affordability. Lastly, executive functioning and MNS should be addressed by specific research, due to their pivotal role in patients’ lives.

## Figures and Tables

**Table 1:** Research strategy.

Database	Research String
PsycINFO	(“Parkinson’s disease” OR “Parkinson” OR “Parkinson’s” OR “Parkinsons” OR “PD” OR “Idiopathic Parkinson’s disease” OR “Idiopathic Parkinson” OR “Idiopathic Parkinson’s” OR “IPD” OR “Parkinson’s disease Idiopathic” OR “Idiopathic Parkinson disease”) AND (“cognitive training” OR “cognitive rehabilitation” OR “cognitive intervention” OR “attention training” OR “brain training” OR “reasoning training” OR “mnemonic training” OR “speed and processing training” OR “executive function* training” OR “mirror neurons” OR “action observation” OR “motor imagery”)
PubMed	(“Parkinson’s disease” OR “Parkinson” OR “Parkinson’s” OR “Parkinsons” OR “PD” OR “Idiopathic Parkinson’s disease” OR “Idiopathic Parkinson” OR “Idiopathic Parkinson’s” OR “IPD” OR “Parkinson’s disease Idiopathic” OR “Idiopathic Parkinson disease”) AND (“cognitive training” OR “cognitive rehabilitation” OR “cognitive intervention” OR “attention training” OR “brain training” OR “reasoning training” OR “mnemonic training” OR “speed and processing training” OR “executive function* training” OR “mirror neurons” OR “action observation” OR “motor imagery”)
Web of science	(TS = (“Parkinson’s disease” OR “Parkinson” OR “Parkinson’s” OR “Parkinsons” OR “PD” OR “Idiopathic Parkinson’s disease” OR “Idiopathic Parkinson” OR “Idiopathic Parkinson’s” OR “IPD” OR “Parkinson’s disease Idiopathic” OR “Idiopathic Parkinson disease”)) AND TS = (“cognitive training” OR “cognitive rehabilitation” OR “cognitive intervention” OR “attention training” OR “brain training” OR “reasoning training” OR “mnemonic training” OR “speed and processing training” OR “executive function* training” OR “mirror neurons” OR “action observation” OR “motor imagery”)

**Table 2:** Included studies’ main information and results.

Source	Study Design	Experimental Treatment	Control Treatment	Results
**Bode et al., 2023 [51]**	Multicentre randomised controlled trials	NEUROvitalis Parkinson training (CT) **Duration:** 6 weeks **Intensity:** twice a week **Type of treatment:** standardised programme targeting executive functions, memory, attention, and visuo-cognition through group and individual tasks. Psycho-educative elements on cognitive functions and strategies to enhance targeted functions were also included in each session.	Low-intensity physical activity programme (PT) **Duration:** 6 weeks **Intensity:** twice a week **Type of treatment:** active control training aimed to improve motor function, but not cognition. Sessions included warm-up exercises, stretching, flexibility, loosening up, and relaxation, as well as psychoeducation and homework.	**Motor outcomes:** CT group displayed more periods of physical activity after training vs. the PT group. **Cognitive outcomes:** CT group: improved EF were related to ↑ active periods and ↓ in active mean bout lengths improved EF ↑ engaging in active behaviours at post-test Both CT and PT groups: ↑ attention with an unstable time effect.
**van Balkom et al., 2022 [57]**	Double-blind randomised controlled trials	Computerised cognitive training **Duration:** 8 weeks **Intensity:** three sessions a week **Type of treatment:** intervention consisted of 13 training games that focused on attention, processing speed and executive functions, and had an adaptive difficulty based on the performance, based on the Braingymmer online CT platform	Active computer-based control group **Duration:** 8 weeks **Intensity:** three sessions a week **Type of treatment:** intervention consisted of three games without difficulty adjustments.	**Cognitive outcomes:** Computerised cognitive training group: no effect on planning task accuracy ↑ processing speed in ToL response time only no other effect on other cognitive domains
**Sousa et al., 2021 [59]**	Randomised controlled trials with placebo	Paper-pencil cognitive training **Duration:** 4 weeks **Intensity:** twice a week **Type of treatment:** group training that emphasised attention and executive dysfunction, plus all the activities of the general rehabilitation programme. Paper-and-pencil tasks focused on the repeated practice of structured exercises. In the same session, three levels of difficulty were offered.	General rehabilitation programme **Duration:** 4 weeks **Intensity:** twice a week **Type of treatment:** various group activities, including: physiotherapy dance, re-education in writing, speech therapy, information groups, manual skills workshops, and physical activity.	**Cognitive outcomes:** Paper-pencil cognitive training: ↑ in attention (especially shifting attention and processing speed), executive functions (verbal fluency) and global measures in the ACE-III battery **Autonomy and QoL outcomes:** Paper-pencil cognitive training: ↑ in QoL
**Vlagsma et al., 2020 [58]**	Multicentre randomised controlled trials	ReSET training **Duration:** 7–14 weeks **Intensity:** once/twice a week **Type of treatment:** individual treatment, to improve or stabilise the participants’ level of independence and QoL, by teaching the patient strategies to compensate for impairments in EF in everyday life situations. Three modules: “Information and awareness”, “Goal setting and planning”, and “Initiative, execution and regulation”.	CogniPlus training **Duration:** 7–14 weeks **Intensity:** once/twice a week **Type of treatment:** six subtests of Cogniplus were individually administered to patients; five subtests aimed at training aspects of attention, and one subtest aimed at training working memory.	**Cognitive outcomes:** RESET training group: immediately after treatment, patients referred to have attained their goals to a larger extent and have experienced fewer executive complaints vs. CogniPlus training group no changes in executive functioning in the long term no changes in other cognitive domains **Social outcomes:** Both groups: no significant effects on the level of participation in societal domains
**De Luca et al., 2019 [43]**	Randomised controlled trials	Computerised cognitive training (CACR) with ERICA platform **Duration:** 8 weeks **Intensity:** three sessions a week **Type of treatment:** training with ERICA, an Italian computerised cognitive tool, comprising a series of specific cognitive exercises.	Standard cognitive training (SCT) **Duration:** 8 weeks **Intensity:** three sessions a week **Type of treatment:** face-to-face interaction between therapist and patient, and paper-and pencil-activities.	**Cognitive outcomes:** CACR group ↑ visual-spatial and executive domains vs. SCT
**Bernini et al., 2019 [44]**	Open not blind randomised controlled trials	CoRe cognitive training + standard physical training (G1) **Duration:** 4 weeks **Intensity:** three sessions a week **Type of treatment:** computer-based logical-executive patient-tailored tasks CoRE and physical rehabilitation. Standard physical rehabilitation comprised cardiovascular warm-up activities, active and passive exercises, stretching, postural changes, and exercises operating on balance and postural control.	Standard physical training (G2) **Duration:** 4 weeks **Intensity:** three sessions a week **Type of treatment:** same standard physical rehabilitation of G1.	**Cognitive outcomes:** G1 group: ↑ MoCA and executive tests vs. G2 Both G1 and G2 groups: no post-training improvement was maintained 6 months later
**Goedeken et al., 2018 [63]**	Single-blind randomised controlled trials	Implementation intention training (II) **Duration:** 3 days **Intensity:** 3 days **Type of treatment:** computer-based prospective memory test. Participants encountered activities for which they made decisions; also, they encountered prospective memory tasks that they had to remember to “perform” another related task.	Verbal rehearsal training (VR) **Duration:** 3 days **Intensity:** 3 days **Type of treatment:** patients recited the prospective memory tasks they encountered aloud at least three times and studied them for 30 s. Participants were instructed to use their strategy as much as possible in their everyday lives to help them remember to do things.	**Cognitive outcomes:** VR group ↓ self-reported everyday prospective memory vs. II group.
**Peña et al., 2014 [66]**	Randomised controlled trials	REHACOP cognitive training **Duration:** 13 weeks **Intensity:** three sessions a week **Type of treatment:** structured group format programme using paper-pencil tasks with a gradual level of cognitive effort and demand. It trained different cognitive domains and included one module for ADL.	Occupational training (active control group) **Duration:** 13 weeks **Intensity:** three sessions a week **Type of treatment:** occupational group activities; including drawing, reading the daily news, and constructing using different materials.	**Cognitive outcomes:** REHACOP group ↑ in visual memory, TOM, functional disability, and processing speed vs. Occupational training group
**Petrelli et al., 2014 [52]**	Randomised controlled trials	1. NEUROvitalis training **Duration:** 6 weeks **Intensity:** twice a week **Type of treatment:** structured training programme that includes individual tasks, group tasks and group games each focusing on specific cognitive functions, and with a corresponding psychoeducational part. 2. Mentally Fit training **Duration:** 6 weeks **Intensity:** twice a week **Type of treatment:** unstructured, not domain-specific “brain jogging” program. Domains were not addressed in focused sessions, individual and group tasks or conversations.	Control waiting list group **Duration:** N.A. **Intensity:** N.A. **Type of treatment:** no training between test sessions.	**Cognitive outcomes:** NEUROvitalis training group: ↑ verbal short-term memory and executive functions (working memory) Mentally Fit training group: no significant cognitive gains ↓ depression scores
**Zimmermann et al., 2014 [68]**	Parallel single-blind randomised controlled trials	CogniPlus training **Duration:** 4 weeks **Intensity:** three sessions a week **Type of treatment:** CogniPlus training programme, specifically aimed to improve focused attention, working memory, executive functions, and inhibition. The level of difficulty was adapted automatically by the program itself, or manually if necessary.	Nintendo Wii exergames training **Duration:** 4 weeks **Intensity:** three sessions a week **Type of treatment:** Nintendo Wii, a game console with movement-capturing controllers. The patients were seated, so that they could not fall. In each session, the patients played four sports games from Wii Sports Resort: Table Tennis, Swordplay, Archery, and Air Sports. The level of difficulty was adapted automatically by the game.	**Cognitive outcomes:** Both groups: ↑ attention, working memory, inhibition, and planning Nintendo Wii exergames training group: ↑ attention vs. CogniPlus training group
**Sammer et al., 2006 [53]**	Randomised controlled trials	Executive functions training **Duration:** 3–4 weeks **Intensity:** 10 sessions **Type of treatment:** cognitive training in which all methods were designed to improve working memory abilities associated with executive functions. Speech production was encouraged by requesting patients to tell short stories. A set of photos was used to train working memory and to produce short stories.	Standard treatment **Duration:** 3–4 weeks **Intensity:** 10 sessions **Type of treatment:** standard training, including: occupational therapy, physiotherapy, and physical treatment.	**Cognitive outcomes:** Executive functions training group: ↑ core executive abilities (rule shift, and organising performance of a task) maintained in the after-treatment measurement Standard treatment: no significant improvement
**Maidan et al., 2017 [69]**	Randomised controlled trials	Treadmill training + Virtual reality (TT + VR) **Duration:** 6 weeks **Intensity:** three sessions a week **Type of treatment:** patients walked on a treadmill while reacting to a virtual environment that included real-life challenges requiring continual adjustment of steps and provided visual and auditory feedback.	Treadmill training (TT) **Duration:** 6 weeks **Intensity:** 3threesessions a week **Type of treatment:** active control intervention in which patients walked on a treadmill, with similar intensity and duration as the experimental group, but without the VR simulation.	**Motor outcome:** TT + VR group ↓ falls incidents **Neurophysiological outcome:** TT + VR group: ↓ activation in inferior frontal gyrus, different patterns of brain activation during imagined obstacle negotiation
**Edwards et al., 2013 [64]**	Randomised trial	Cognitive speed of processing training (SOPT) **Duration:** 20 h **Intensity:** three sessions a week **Type of treatment: A** self-administered version of SOPT, InSight, was completed by participants at home. InSight included five exercises designed to improve information processing speed in realistic visual contexts and four additional exercises.	Control waiting list group **Duration:** N.A. **Intensity:** N.A. **Type of treatment:** no contact.	**Cognitive outcomes:** SOPT group: ↑ UFOV performance no results in immediate improvements in either cognitive self-perceptions or depressive symptoms
**Pompeu et al., 2012 [60]**	Parallel prospective single-blind randomised clinical trial	Wii-based exergames cognitive and motor training **Duration:** 7 weeks **Intensity:** twice a week and an additional session was performed 60 days after the end of training. **Type of treatment:** balance training by playing 10 Wii Fit games. The cognitive demands of the games were attention to solving the tasks, working memory and performance management.	Active balance control group **Duration:** 7 weeks **Intensity:** twice a week **Type of treatment:** balance exercise therapy developed considering the games chosen for the experimental group. The control group performed balance exercises that were equivalent to the motor demands of the experimental group but without the provision of external cues, feedback and cognitive stimulation.	**Motor outcomes:** Both groups: ↑ balance maintained 60 days after training end no improvements in balance in the dual-task **Cognitive outcomes:** Both groups: ↑ global cognition maintained 60 days after training ends **Autonomy and QoL outcomes:** Both groups: ↑ ADL maintained 60 days after training ends
**Leocadi et al., 2024 [45]**	Randomised clinical/fMRI study	DUAL-TASK + AOT-MI training **Duration:** 6 weeks **Intensity:** Not specified **Type of treatment:** gait/balance training consisting of AOT and MI in combination with observed-imagined exercises.	DUAL-TASK training **Duration:** 6 weeks **Intensity:** Not specified **Type of treatment:** participants performed the same number of exercises as the experimental group combined with watching landscape videos instead of observation/imagination.	**Cognitive outcomes:** Both groups: ↑ accuracy in a task relying on set-shifting (specific for the attentive–executive domain) DUAL-TASK + AOT-MI group: no specific effect on cognition **Neurophysiological outcomes:** DUAL-TASK + AOT-MI group: ↑ substantial brain functional changes vs. DUAL-TASK group
**Maggio et al., 2021 [46]**	Randomised clinical study	1. Tele-VR cognitive training (EG1) **Duration:** 6 weeks **Intensity:** three sessions a week **Type of treatment:** remote programme using two cognitive rehabilitation apps on smartphones; that offered science-based brain training enhancing cognitive performance across multiple cognitive domains. 2. Tele-VR cognitive and socio-cognitive training (EG2) **Duration:** 6 weeks **Intensity:** three sessions a week **Type of treatment:** remote program via one cognitive rehabilitation app, and one social-cognitive rehabilitation app; in which the patient overcame social challenges with audiovisual feedback.	Not-VR cognitive training (aCG) **Duration:** 6 weeks **Intensity:** three sessions a week **Type of treatment:** conventional training conducted using paper-pencil exercises performed independently at home and evaluated by the therapist at the end of the rehabilitation programme. Worksheets containing cognitive exercises, targeting both cognitive and emotional-social components and including various types of exercises were used.	**Cognitive outcomes:** Both EG1 and EG2 groups: ↑ in the subjective perception of memory performance, MoCA and FAB scores vs. aCG group EG2 group: ↑ MoCA and FAB scores vs. aCG group aCG group: ↑ executive-attentive and visuospatial domains **Socio-emotional outcomes:** Both EG1 and EG2 groups: ↑ mood and TOM vs. aCG group
**Gobbi et al., 2021 [61]**	Randomised controlled trials with crossover features	1. Multimodal training **Duration:** 32 weeks **Intensity:** twice a week **Type of treatment:** training for improving/maintaining all components of functional capacity. Individuals enrolled in the Multimodal exercise group in the first year were switched to the Functional Mobility or Mental/Leisure group in the second year, and Mental/Leisure or Functional Mobility for the third year. 2. Functional Mobility training **Duration:** 32 weeks **Intensity:** twice a week **Type of treatment:** training to improve/maintain balance and locomotion parameters as well as functional capacity and participants’ QoL.	Mental/Leisure training **Duration:** 32 weeks **Intensity:** twice a week **Type of treatment:** cognitive and leisure activities. This programme included two periods, including three sub-periods each. The sub-periods, based on different leisure dimensions (social, manual, and artistic), were always combined with intellectual and social aspects, such as social activities, math problem-solving, card and memory games, drawing, debates, and lectures.	**Cognitive outcomes:** Multimodal training group: ↑ executive function, attention, and working memory vs. Functional Mobility training and Mental/Leisure training groups not have any substantial benefits on executive functions at the 8 months follow-up not able to delay the progressive decline in cognitive functions at the 8 month follow-up **Emotional outcomes:** Multimodal training group: ↓ physical stress
**Bernini et al., 2021 [47]**	Three arm double-blind randomised controlled trials	CoRe cognitive training (CCT) **Duration:** 3 weeks **Intensity:** four sessions a week **Type of treatment:** CoRe, a software tool, administered 11 tasks targeting several cognitive abilities. These tasks were computerised versions of existing paper-and-pencil exercises or were created to meet specific requirements. The individual patient’s performance was analysed to set the appropriate difficulty level which progressively increased.	1. Paper-pencil cognitive training (PCT) **Duration:** 3 weeks **Intensity: four** sessions a week **Type of treatment:** same training programme as the CCT group but using the paper-and-pencil version of the tasks. The increasing levels of difficulty were managed by the therapist. 2. Unstructured activity training (CG) **Duration:** 3 weeks **Intensity:** four sessions a week **Type of treatment:** unstructured activities that served as a behavioural placebo treatment.	**Cognitive outcomes:** CCT group: ↑ MoCA scores, attention, and processing speed domains vs. PCT and CG groups PCT group: ↑ attention/processing speed domain vs. CG group
**Mariano Barboza et al., 2019 [62]**	Randomised clinical trial	Cognitive–Motor training (CMG) **Duration:** 16 weeks **Intensity:** twice a week **Type of treatment:** intervention performed in two parts: the same protocol used in the MG and, at the end of each therapy session, 30 min of cognitive paper-pencil tasks with gradually increased difficulty. The participants received three more activities to perform at home, which were reviewed in the next session.	Motor training (MG) **Duration:** 16 weeks **Intensity:** twice a week **Type of treatment:** protocol focused on balance training, sensory integration, agility and motor coordination, exploration of limits of stability, anticipatory and reactive postural adjustments, functional independence, and gait improvement. The therapy sessions were divided into four blocks with a gradual increase in exercise complexity.	**Cognitive outcomes:** Both CMG and MG groups: ↑ short-term memory and visuospatial function
**Alloni et al., 2018 [48]**	Single-blind randomised controlled trials	CoRe cognitive training (G1) **Duration:** 4 weeks **Intensity:** three sessions a week **Type of treatment:** CoRe system (Cognitive Rehabilitation); a software tool that automatically generates patient-tailored exercises using a big set of stimuli organised into an ontology.	Sham training (G2) **Duration:** 4 weeks **Intensity:** three sessions a week **Type of treatment:** only sham intervention; no cognitive training.	**Cognitive outcomes:** G1 group: ↑ executive and memory functions vs. G2 (not maintained after the discharge)
**Lawrence et al., 2018 [70]**	Randomised controlled trials	1. Tailored cognitive training **Duration:** 4 weeks **Intensity:** three sessions a week **Type of treatment:** patients completed individualised activities on Smartbrain Pro; an interactive computer-based training programme designed to train each cognitive domain. Performance was automatically monitored by the programme to adjust individual difficulty levels for each activity. 2. Tailored cognitive training + tDCS **Duration:** 4 weeks **Intensity:** 3 sessions a week + tDCS once a week **Type of treatment:** same as tailored cognitive training, plus 20 min of tDCS.	1. Standard cognitive training **Duration:** 4 weeks **Intensity:** three sessions a week **Type of treatment:** computer-based training. Predetermined programme comprising 10 activities, two activities per cognitive domain 2. Standard cognitive training + tDCS **Duration:** 4 weeks **Intensity:** three sessions a week + tDCS once a week **Type of treatment:** same as standard cognitive training, plus 20 min of stimulation. 3. Only tDCS **Duration:** 4 weeks **Intensity:** once a week **Type of treatment:** 20 min of stimulation. 4. Waiting list **Duration:** N.A. **Intensity:** N.A. **Type of treatment:** participants completed baseline, post-intervention, and 12-week follow-up neuropsychological assessments but did not complete cognitive training or tDCS.	**Cognitive outcomes:** Standard cognitive training group: ↑ memory Tailored cognitive training group: ↑ attention and working memory tDCS group: ↑ attention, working memory and memory Standard cognitive training + tDCS group: ↑ executive function, attention and working memory Tailored cognitive training + tDCS group: ↑ executive function, attention/working memory, and memory **Autonomy and QoL outcomes:** Standard cognitive training group: ↑ ADL and QoL Tailored cognitive training group: ↑ QoL Standard cognitive training + tDCS group: ↑ ADL
**Kalbe et al., 2020 [54]**	Multicentre randomised controlled trials	NEUROvitalis Parkinson training (CT) **Duration:** 6 weeks **Intensity:** twice a week **Type of treatment:** intervention targeting executive functions, memory, attention, and visuocognition. Each session is characterised by several training elements: psychoeducation group tasks and activity games, individual exercises, and homework.	Low-intensity physical activity training (CG) **Duration:** 6 weeks **Intensity:** twice a week **Type of treatment:** intervention aimed to be beneficial for PD patients but to have minimal effects on cognition. The main trained domains are stretching, flexibility, loosening up, and relaxation; also, psychoeducation on PD symptoms, therapy options and homework were conducted.	**Cognitive outcomes:** CT group: ↑ executive functions (especially verbal fluency), but not memory CG group: ↑ working memory
**Reuter et al., 2012 [55]**	Blind randomised study	Cognitive, transfer, and psychomotor training (Group C): **Duration:** 3–4 weeks + prosecution at home **Intensity:** four cognitive training sessions a week + three transfer training sessions a week + three psychomotor training sessions a week + three cognitive training sessions a week at home, two transfer training sessions a week and two psychomotor training sessions a week. **Type of treatment:** cognitive training in addition to transfer and psychomotor training, with a prosecution at home. The cognitive training employed a computer-based programme, and it included training of different cognitive functions. For transfer training, patients were asked to practise competence in tasks of daily routines. Psychomotor training included games and tasks designed to learn how to perform motor sequences. Also, mental imagery and aerobic training were employed.	1. Transfer and cognitive training (Group B) **Duration:** 3–4 weeks + prosecution at home **Intensity:** four cognitive training sessions a week + three transfer training sessions a week + three cognitive training sessions a week at home, two transfer training sessions a week, and two relaxation training a week. **Type of treatment:** same cognitive and transfer training as Group C, with a prosecution at home. While at home, instead of the same psychomotor training of Group C, participants performed relaxation training. 2. Only cognitive training (Group A) **Duration:** 3–4 weeks + prosecution at home **Intensity:** four cognitive training sessions a week + three cognitive training sessions a week at home, two transfer training sessions a week, and two relaxation training a week. **Type of treatment:** same cognitive training of Group C and B, with a prosecution at home.	**Cognitive outcomes:** Group C: ↑ cognitive performance vs. Group B and A Group B: ↑ cognitive performance vs. Group A
**Prat Paris et al., 2011 [67]**	Blind multicentre randomised controlled trials	Cognitive training (CTG) **Duration:** 4 weeks **Intensity:** three sessions a week **Type of treatment:** interactive multimedia software and paper-and-pencil exercises. Computer-aided training employed the SmartBrain tool, designed to stimulate specific and non-specific cognitive domains. Participants received a pack with 20 cognitive homework exercises designed to stimulate specific and nonspecific cognitive areas.	Speech therapy **Duration:** 4 weeks **Intensity:** three times a week group sessions + once-a-week individual tutored session **Type of treatment:** speech therapy aimed to make participants aware of their speech and communication difficulties.	**Cognitive outcomes:** CTG group: ↑ in attention, information processing speed, memory, visuospatial and visuoconstructive abilities, semantic verbal fluency, and executive functions vs. Speech therapy group **Autonomy and QoL outcomes:** CTG group: no significant improvements in QoL
**Sarasso et al., 2021 [49]**	Randomised clinical/fMRI study	DUAL-TASK + AOT-MI training **Duration:** 6 weeks **Intensity:** three sessions a week **Type of treatment:** patients performed a gait/balance training consisting of AOT-MI combined with practising the observed-imagined exercises	DUAL-TASK training **Duration:** 6 weeks **Intensity:** three sessions a week **Type of treatment:** patients performed the same number of exercises combined with watching landscape videos instead of observation/imagination, and exercises were increasingly difficult, including the dual-task.	**Motor outcomes:** Both groups: ↑ in mobility during TUG-COG, TUG-MAN, and TUG (maintained 2 months after training) DUAL-TASK + AOT-MI group: ↑ change in TUG-COG mean, and peak of turning velocity during TUG and TUG-COG (maintained at follow-up) vs. DUAL-TASK group
**Agosta et al., 2017 [50]**	Prospective randomised study	AOT Group **Duration:** 4 weeks **Intensity:** three sessions a week **Type of treatment:** physical therapy training: during each training session, two video clips showing strategies useful in circumventing FoG episodes, were presented twice. Overall, subjects in the AOT group were presented with six video clips, repeated each week. The complexity of actions increased, and auditory cues were associated with the movements. After each video clip observation, patients were asked to imitate the observed actions repetitively and accurately at the beat of the auditory cues.	Landscape Group **Duration:** 4 weeks **Intensity:** three sessions a week **Type of treatment:** physical therapy training, during each training session they watched video clips containing sequences of static pictures of landscapes without any living representations for the same time length. During training sessions, patients performed the same movements/actions used for the AOT group in the same order and amount of time, following the physical therapist’s instructions.	**Motor outcomes:** Both groups: ↓ FoG severity ↑ walking speed AOT group: ↓ motor impairment (after short-term follow-up) vs. Landscape group ↑ walking speed and balance (after short-term follow-up) vs. Landscape group **Autonomy and QoL outcomes:** Both groups: ↑ quality of life AOT group: ↑ quality of life vs. Landscape group (after short-term follow-up)
**Suarez-Garcia et al., 2021 [65]**	Randomised blinded sham-controlled study	PD-atDCS Group **Duration:** 5 consecutive days **Intensity:** three phases **Type of treatment:** three phases of a protocol with stimulation; pre-stimulation phase, stimulation phase, and post-stimulation phase. Both before and after the stimulation protocol, participants completed a PWA task involving action-verb and object-noun conditions. During the stimulation phase, participants received 20 min of online stimulation while completing a cognitive training protocol. The post-stimulation phase was identical in structure and duration to the pre-stimulation phase.	PD-stDCS Group **Duration:** 5 consecutive days **Intensity:** three phases **Type of treatment:** same three phases of the experimental treatment; except for receiving a sham stimulation, lasting 1 min and not effective.	**Cognitive outcomes:** PD-atDCS group: ↑ for action-verb processing vs. PD-stDCS group no effect on object-noun processing
**Vriend et al., 2021 [56]**	Double-blind randomised controlled trials	Computerised cognitive training **Duration:** 8 weeks **Intensity:** three sessions a week **Type of treatment:** home-based computer intervention employed 13 training games with adaptive difficulty that focused on different cognitive functions and were adapted from the Braingymmer online platform.	Computerised active control group **Duration:** 8 weeks **Intensity:** three sessions a week **Type of treatment:** home-based, computer intervention that employed three low-threshold games with constant difficulty primarily based on “crystallised intelligence” factors, i.e., solitaire, hangman, and trivia questions.	**Cognitive outcomes:** Computerised cognitive training group: faster responses on the ToL task vs. Computerised active control group **Neurophysiological outcomes:** Computerised cognitive training group: no effect on network topology neither on the global or subnetwork level

Note: Full information regarding included studies’ materials and methods are reported in Supplementary Materials, Table S1. Abbreviations in alphabetical order: ↑: increasing in the outcome; ↓: decreasing in the outcome; ACE-III: Addenbrooke Cognitive Examination-III; aCG: active Control Group; ADL: Activities of Daily Living; AOT: Action Observation Training; AOT-MI: Action Observation Training-Motor Imagery; atDCS: anodal transcranial Direct Current Stimulation; CACR: computer-assisted cognitive rehabilitation; CCT: Computer-based cognitive training; CG: Control Group; CMG: Cognitive motor group; CoRE: Cognitive Rehabilitation computer software; CT: Cognitive training; CTG: cognitive training group; EF: executive functions; EG1: Experimental Group 1; EG2: Experimental Group 2; FAB: Frontal Assessment Battery; fMRI: functional Magnetic Resonance Imaging; FoG: Freezing of Gait; G1: Group 1; G2: Group 2; II: Implementation Intention; MG: Motor group; MoCA: Montreal Cognitive Assessment; N.A.: not applicable; PCT: Paper-pencil cognitive training; PD: Parkinson’s disease; PD-atDCS Group: Parkinson’s disease-anodal transcranial Direct Current Stimulation Group; PD-stDCS Group: Parkinson’s disease-sham transcranial Direct Current Stimulation Group; PRMQ-Pro: Prospective and Retrospective Memory Questionnaire-Pro; PT: Physical training; QoL: quality of life; REHACOP: cognitive rehabilitation programme in psychosis; ReSET training: Strategic Executive Treatment; RRL: The Role Resumption list; SCT: standard cognitive training; SOPT: speed of processing training; stDCS: sham transcranial Direct Current Stimulation; tDCS: transcranial Direct Current Stimulation; ToL: Tower of London; TOM: theory of mind; TT: Treadmill training; TT + VR: Treadmill training + Virtual Reality; TUG: Timed-Up-and–Go test; TUG-COG: Timed-Up-and–Go test-Cognitive; TUG-MAN: Timed-Up-and–Go test-Manual task; UFOV: Useful Field of View Test; VR: Verbal Rehearsal.

## Data Availability

All relevant data generated and analysed are included in the present study.

## Supplementary Materials

The following supporting information can be downloaded at: https://www.mdpi.com/article/10.3390/brainsci15010061/s1, File S1. PRISMA checklist, Table S1 Studies’ design and method information, Table S2. Studies and sample characteristics.
