# Supplementary material for: Identification of Cognitive Training for Individuals with Parkinson’s Disease: A Systematic Review
Source: Brain Sci. 2025 Jan 11;15(1):61. doi: 10.3390/brainsci15010061 (PMC11763612; doi:10.3390/brainsci15010061)
Supplement: Supplementary file 1 [file brainsci-15-00061-s001.zip › Table S1.Studies’ design and method information.pdf]

Supplementary Materials – Table S1. Studies' design and method information

Source	Study Design	Experimental Treatment	Control Treatment	Main Outcome	Secondary Outcomes	Results
**Bode et al., 2023**	Multicentre randomised controlled trials	NEUROvitalis Parkinson training (CT)<br>**Duration:** 6 weeks<br>**Intensity:** twice a week<br>**Type of treatment:** standardised program targeting executive functions, memory, attention, and visuo-cognition through group and individual tasks. Psycho-educative elements on cognitive functions and strategies to enhance targeted functions were also included in each session.	Low-intensity physical activity program (PT)<br>**Duration:** 6 weeks<br>**Intensity:** twice a week<br>**Type of treatment:** active control training aimed to improve motor function, but not cognition. Sessions included warm-up exercises, stretching, flexibility, loosening up, and relaxation, as well as psychoeducation and homework.	**Duration of activity:**<br>-total duration spent in physical activity or sedentary behaviour<br>-number of active or sedentary periods<br>-TEE (in kcal) in physical activity or sedentary behaviour<br>-mean bout lengths spent in physical activity or sedentary behaviour	**Memory:**<br>-CVLT-II recognition and dr<br>-ROCFT-dr<br>**Executive functions:**<br>-Semantic and phonemic word fluency<br>-MCST categories<br>-Key search<br>**Attention:**<br>-d2-R errors and concentration performance<br>**Working memory:**<br>WAIS (letter-number sequencing, digit span backward)<br>**Visuocognition:**<br>-ROCFT-copy<br>-Benton judgment of line orientation<br>**Language:**<br>-BNT<br>-ACL<br>(Taken from Kalbe et al., 2020)	**Motor outcomes:**<br>CT group displayed more periods of physical activity after training *vs.* PT group.<br>**Cognitive outcomes:**<br><u>CT group:</u><br>•improved EF were related to ↑ active periods and ↓ in active mean bout lengths<br>•improved EF ↑ engaging in active behaviours at post-test<br><u>Both CT and PT groups:</u><br>↑ attention with an unstable time effect.
**van Balkom et al., 2022**	Double-blind randomised controlled trials	Computerised cognitive training<br>**Duration:** 8 weeks<br>**Intensity:** 3 sessions a week<br>**Type of treatment:**	Active computer-based control group<br>**Duration:** 8 weeks<br>**Intensity:** 3 sessions a week<br>**Type of treatment:**	**Planning task accuracy:**<br>ToL accuracy	**ToL response time**<br>**Subjective cognitive complaints:**<br>-PD-CFRS inf<br>-PD-CFRS<br>-CFQ	**Cognitive outcomes:**<br><u>Computerised cognitive training group</u>:<br>•no effect on planning task accuracy

Source	Study Design	Experimental Treatment	Control Treatment	Main Outcome	Secondary Outcomes	Results
		intervention consisted of 13 training games that focused on attention, processing speed and executive functions, and had an adaptive difficulty based on the performance, based on the Braingymmer online CT platform	intervention consisted of 3 games without difficulty adjustments.		**Latent cognitive factors**<br>**Global cognition:** MoCA	•↑ processing speed in ToL response time only<br>•no other effect for other cognitive domains
**Sousa et al., 2021**	Randomised controlled trials with placebo	Paper-pencil cognitive training<br>**Duration:** 4 weeks<br>**Intensity:** twice a week<br>**Type of treatment:** group training that emphasised attention and executive dysfunction, plus all the activities of the general rehabilitation program. Paper-and-pencil tasks focused on the repeated practice of structured exercises. In the same session, three levels of difficulty were offered.	General rehabilitation program<br>**Duration:** 4 weeks<br>**Intensity:** twice a week<br>**Type of treatment:** various group activities, including: physiotherapy dance, re-education in writing, speech therapy, information groups, manual skills workshops, and physical activity.	**Cognitive functions:**<br>-Digit Span<br>-TMT-A<br>-TMT-B<br>-short battery ACE-III	**QoL:**<br>PDQ-39	**Cognitive outcomes:**<br>Paper-pencil cognitive training:<br>↑ in attention (especially shifting attention and processing speed), executive functions (verbal fluency) and global measures in the ACE-III battery<br>**Autonomy and QoL outcomes:**<br>Paper-pencil cognitive training:<br>↑ in QoL
**Vlagsma et al., 2020**	Multicentre randomised controlled trials	ReSET training<br>**Duration:** 7-14 weeks<br>**Intensity:** once/twice a week<br>**Type of treatment:** individual treatment, to improve or stabilise the participants' level of	CogniPlus training<br>**Duration:** 7-14 weeks<br>**Intensity:** once/twice a week<br>**Type of treatment:** 6 subtests of Cogniplus were individually administered to patients; five subtests aimed at training aspects of attention, and one	**Level of participation in societal domains:**<br>RRL	**Executive functions:**<br>-TMT B/A<br>-Visual Elevator subtest of TEA<br>-BADS and Complex Zoo map subtest<br>**Attention and memory functions:**	**Cognitive outcomes:**<br>RESET training group:<br>•immediately after treatment, patients referred to have attained their goals to a larger extent and to have experienced fewer executive complaints *vs.* CogniPlus training group

Source	Study Design	Experimental Treatment	Control Treatment	Main Outcome	Secondary Outcomes	Results
		independence and QoL, by teaching patient strategies to compensate for impairments in EF in everyday life situations.<br>3 modules: "Information and awareness", "Goal setting and planning", "Initiative, execution and regulation".	subtest aimed at training working memory.		-TMT-A<br>-RAVLT<br>**Rating of goal attainment:**<br>TGA	•no changes in executive functioning in the long term<br>•no changes in other cognitive domains<br>**Social outcomes:**<br><u>Both groups:</u><br>no significant effects on level of participation in societal domains
**De Luca et al., 2019**	Randomised controlled trials	Computerised cognitive training (CACR) with ERICA platform<br>**Duration:** 8 weeks<br>**Intensity:** 3 sessions a week<br>**Type of treatment:** training with ERICA, an Italian computerised cognitive tool, comprising a series of specific cognitive exercises.	Standard cognitive training (SCT)<br>**Duration:** 8 weeks<br>**Intensity:** 3 sessions a week<br>**Type of treatment:** face-to-face interaction between therapist and patient and paper and pencil activities.	**Mild cognitive impairment; global and cognitive domains:**<br>-ACE-R AO<br>-ACE-R VS<br>-ACE-R L<br>-ACE-R F<br>-ACE-R M	**Frontal abilities:**<br>-WEIGL<br>-FAB<br>**Anxiety:**<br>HRS-A<br>**Depression:**<br>GDS	**Cognitive outcomes:**<br>CACR group ↑ visual-spatial and executive domains *vs.* SCT
**Bernini et al., 2019**	Open not blind randomised controlled trials	CoRe cognitive training + standard physical training (G1)<br>**Duration:** 4 weeks<br>**Intensity:** 3 sessions a week<br>**Type of treatment:** computer-based logical-executive patient-tailored tasks CoRE and physical rehabilitation.	Standard physical training (G2)<br>**Duration:** 4 weeks<br>**Intensity:** 3 sessions a week<br>**Type of treatment:** same standard physical rehabilitation of G1.	**Global cognitive function:**<br>-MMSE<br>-MoCA	**Logical-executive functions:**<br>-RM47<br>-WEIGL<br>-FAB<br>-FAS	**Cognitive outcomes:**<br><u>G1 group:</u> ↑ MoCA and executive tests *vs.* G2<br><u>Both G1 and G2 groups:</u> no post-training improvement was maintained 6 months later

Source	Study Design	Experimental Treatment	Control Treatment	Main Outcome	Secondary Outcomes	Results
		Standard physical rehabilitation comprised cardiovascular warm-up activities, active and passive exercises, stretching, postural changes, and exercises operating on balance and postural control.				
**Goedeken et al., 2018**	Single-blind randomised controlled trials	Implementation intention training (II)<br>**Duration:** 3 days<br>**Intensity:** 3 days<br>**Type of treatment:** computer-based prospective memory test. Participants encountered activities for which they made decisions; also, they encountered prospective memory tasks that they had to remember to "perform" another related task.	Verbal rehearsal training (VR)<br>**Duration:** 3 days<br>**Intensity:** 3 days<br>**Type of treatment:** patients recited the prospective memory tasks they encounter aloud at least three times and studied them for 30 seconds. Participants were instructed to use their strategy as much as possible in their everyday lives to help them remember to do things.	**Reported Everyday Prospective Memory:**<br>-PRMQ-Pro<br>-PRMQ subscale Pro-Self<br>-PRMQ subscale Pro-Env	**Characteristics Associated with Everyday Prospective Memory Change:**<br>-CEQ<br>-Prospective memory-related awareness (metacognitive knowledge, on-line awareness)<br>-Everyday prospective memory strategy use<br>-MoCA	**Cognitive outcomes:**<br>VR group ↓ self-reported everyday prospective memory *vs.* II group.
**Peña et al., 2014**	Randomised controlled trials	REHACOP cognitive training<br>**Duration:** 13 weeks<br>**Intensity:** 3 sessions a week<br>**Type of treatment:** structured group format program using paper-pencil tasks with a gradual level of cognitive effort and demand. It	Occupational training (active control group)<br>**Duration:** 13 weeks<br>**Intensity:** 3 sessions a week<br>**Type of treatment:** occupational group activities; including drawing, reading the daily news, and constructing using different materials.	**Processing speed:**<br>-TMT-A<br>-Salthouse Letter<br>**Verbal memory:**<br>Hopkins Verbal Learning Test learning and dr<br>**Visual memory:**<br>Brief Visual Memory Test learning and dr	**Depressive symptoms:**<br>GDS<br>**Neuropsychiatric symptoms:**<br>NPI-Q14<br>**Apathy:**<br>Lille Apathy Rating Scale<br>**Functional disability:**	**Cognitive outcomes:**<br>REHACOP group ↑ in visual memory, TOM, functional disability, and processing speed *vs.* Occupational training group

Source	Study Design	Experimental Treatment	Control Treatment	Main Outcome	Secondary Outcomes	Results
		trained different cognitive domains and included one module for ADL.		**Executive functioning:** SCWT<br>**TOM:** Happé test	WHO-DAS II	
**Petrelli et al., 2014**	Randomised controlled trials	1.NEUROvitalis training<br>**Duration:** 6 weeks<br>**Intensity:** twice a week<br>**Type of treatment:** structured training program that includes individual tasks, group tasks and group games each focusing on specific cognitive functions, and with a corresponding psychoeducational part.<br>2.Mentally Fit training<br>**Duration:** 6 weeks<br>**Intensity:** twice a week<br>**Type of treatment:** unstructured, not domain-specific "brain jogging" program. Domains were not addressed in focused sessions, individual and group tasks or conversations.	Control waiting list group<br>**Duration:** N.A.<br>**Intensity:** N.A.<br>**Type of treatment:** no training between test sessions.	**Attention:** Brief test of attention<br>**Verbal memory short- and long-term:**<br>-DemTect<br>-Memo<br>**Visual memory long-term:** Complex figure-dr<br>**Executive functions**<br>-DemTect<br>-Digit span reverse<br>-Semantic fluency (DemTect)<br>-FAS	**Visuo-construction:** Complex figure-ir<br>**Depression:** BDI-II<br>**QoL:** PDQ-39	**Cognitive outcomes:**<br><u>NEUROvitalis training group:</u><br>↑ verbal short-term memory and executive functions (working memory)<br><u>Mentally Fit training group:</u><br>•no significant cognitive gains<br>•↓ depression scores
**Zimmermann et al., 2014**	Parallel single-blind randomised controlled trials	CogniPlus training<br>**Duration:** 4 weeks<br>**Intensity:** 3 sessions a week<br>**Type of treatment:** CogniPlus training program, specifically	Nintendo Wii exergames training<br>**Duration:** 4 weeks<br>**Intensity:** 3 sessions a week<br>**Type of treatment:** Nintendo Wii, a game console with movement-capturing	**Alertness:** Tests of Attentional Performance<br>**Working memory:** Tests of Attentional Performance	**Depression:** BDI-II	**Cognitive outcomes:**<br><u>Both groups:</u><br>↑ attention, working memory, inhibition, and planning<br><u>Nintendo Wii exergames training group:</u>

Source	Study Design	Experimental Treatment	Control Treatment	Main Outcome	Secondary Outcomes	Results
		aimed to improve focused attention, working memory, executive functions, and inhibition. The level of difficulty was adapted automatically by the program itself, or manually if necessary.	controllers. The patients were seated, so that they could not fall. In each session, the patients played 4 sports games from Wii Sports Resort: Table Tennis, Swordplay, Archery, and Air Sports.<br>The level of difficulty was adapted automatically by the game.	**Executive functions:**<br>TMT<br>**Visuoconstruction:**<br>Block-Design Test, (WAIS)<br>**Episodic memory:**<br>CVLT		↑ attention vs. CogniPlus training group
**Sammer et al., 2006**	Randomised controlled trials	Executive functions training<br>**Duration:** 3-4 weeks<br>**Intensity:** 10 sessions<br>**Type of treatment:** cognitive training in which all methods were designed to improve working memory abilities associated with executive functions.<br>Speech production was encouraged by requesting patients to tell short stories. A set of photos was used to train working memory and to produce short stories.	Standard treatment<br>**Duration:** 3-4 weeks<br>**Intensity:** 10 sessions<br>**Type of treatment:** standard training, including: occupational therapy, physiotherapy, physical treatment.	**Executive functions:**<br>-BADS<br>-TKS<br>-ZVT<br>**Working memory:**<br>GNL<br>**Attention:**<br>AKT	**Well-being:**<br>Bf-S<br>**Intelligence:**<br>MWT<br>**Depression:**<br>HAMD	**Cognitive outcomes:**<br><u>Executive functions training group:</u><br>↑ core executive abilities (rule shift, and organising performance of a task) maintained in the after-treatment measurement<br><u>Standard treatment:</u><br>no significant improvement
**Maidan et al., 2017**	Randomised controlled trials	Treadmill training + Virtual reality (TT+VR)<br>**Duration:** 6 weeks<br>**Intensity:** 3 sessions a week<br>**Type of treatment:**	Treadmill training (TT)<br>**Duration:** 6 weeks<br>**Intensity:** 3 sessions a week<br>**Type of treatment:** active control intervention in which patients walked on a treadmill, with similar intensity and	**Brain activation:**<br>imagined walking fMRI task.<br>**Motor imagery abilities:**<br>-chronometric test<br>-KVIQ	**Gait performance:**<br>PKMAS sensorized mat<br>**Cognitive functions:**<br>MoCA	**Motor outcome:**<br>TT+VR group ↓ falls incidents<br>**Neurophysiological outcome:**<br><u>TT+VR group:</u><br>• ↓ activation in inferior frontal gyrus

Source	Study Design	Experimental Treatment	Control Treatment	Main Outcome	Secondary Outcomes	Results
		patients walked on a treadmill while reacting to a virtual environment that included real-life challenges requiring continual adjustment of steps and provided visual and auditory feedback.	duration as the experimental group, but without the VR simulation.		**Global cognitive function, attention, executive function:** Computerized Mindstreams battery	•different patterns of brain activation during imagined obstacle negotiation
**Edwards et al., 2013**	Randomized trial	Cognitive speed of processing training (SOPT)<br>**Duration:** 20 hours<br>**Intensity:** 3 sessions a week<br>**Type of treatment: A** self-administered version of SOPT, InSight, was completed by participants at home. InSight included 5 exercises designed to improve information processing speed in realistic visual contexts and 4 additional exercises.	Control waiting list group<br>**Duration:** N.A.<br>**Intensity:** N.A.<br>**Type of treatment:** no contact.	**Cognitive speed of processing:** UFOV	**Cognitive self-perceptions:** Cognitive Self-Report Questionnaire<br>**Depressive symptoms:** CES-D short form	**Cognitive outcomes:**<br><u>SOPT group:</u><br>•↑ UFOV performance<br>•no results in immediate improvements in either cognitive self-perceptions or depressive symptoms
**Pompeu et al., 2012**	Parallel prospective single-blind randomised clinical trial	Wii-based exergames cognitive and motor training<br>**Duration:** 7 weeks<br>**Intensity:** twice a week and an additional session was performed 60 days after the end of training.<br>**Type of treatment:**	Active balance control group<br>**Duration:** 7 weeks<br>**Intensity:** twice a week<br>**Type of treatment:** balance exercise therapy developed considering the games chosen for the experimental group. The control group performed balance exercises that were	**Independent performance of ADL:** UPDRS-II	**Dynamic balance:** Berg Balance Scale<br>**Static balance:** Unipedal Stance Test<br>**Automatic control:** Unipedal Stance Test performed as a dual task (concurrent with verbal fluency)	**Motor outcomes:**<br><u>Both groups:</u><br>•↑ balance maintained 60 days after training end<br>•no improvements in balance in the dual task<br>**Cognitive outcomes:**<br><u>Both groups:</u><br>↑ global cognition maintained 60 days after training end

Source	Study Design	Experimental Treatment	Control Treatment	Main Outcome	Secondary Outcomes	Results
		balance training by playing 10 Wii Fit games. The cognitive demands of the games were attention to solving the tasks, working memory and performance management.	equivalent to the motor demands of the experimental group but without the provision of external cues, feedback and cognitive stimulation.		**Cognitive performance:**<br>MoCA	**Autonomy and QoL outcomes:**<br>Both groups:<br>↑ ADL maintained 60 days after training end
**Leocadi et al., 2024**	Randomised clinical/ fMRI study	DUAL-TASK + AOT-MI training<br>**Duration:** 6 weeks<br>**Intensity:** Not specified<br>**Type of treatment:** gait/balance training consisting of AOT and MI in combination with observed-imagined exercises.	DUAL-TASK training<br>**Duration:** 6 weeks<br>**Intensity:** Not specified<br>**Type of treatment:** participants performed the same number of exercises as the experimental group combined with watching landscape videos instead of observation/ imagination.	**Resting-state functional connectivity (RS-FC):**<br>fMRI scans	**Multidomain cognition:**<br>CANTAB sub-tests (MOT, AST, OTS, SRM, SWM)	**Cognitive outcomes:**<br>Both groups:<br>↑ accuracy in a task relying on set-shifting (specific for the attentive–executive domain)<br>DUAL-TASK + AOT-MI group:<br>no specific effect on cognition<br>**Neurophysiological outcomes:**<br>DUAL-TASK + AOT-MI group:<br>↑ substantial brain functional changes *vs.* DUAL-TASK group
**Maggio et al., 2024**	Randomised clinical study	1.Tele-VR cognitive training (EG1)<br>**Duration:** 6 weeks<br>**Intensity:** 3 sessions a week<br>**Type of treatment:** remote program using two cognitive rehabilitation apps on smartphones; that offered science-based brain training enhancing cognitive performance across multiple cognitive domains.	Not-VR cognitive training (aCG)<br>**Duration:** 6 weeks<br>**Intensity:** 3 sessions a week<br>**Type of treatment:** conventional training conducted using paper-pencil exercises performed independently at home and evaluated by the therapist at the end of the rehabilitation program. Worksheets containing cognitive exercises, targeting both cognitive and emotional-social components	**Cognitive outcomes:**<br>-MoCA<br>-MMSE<br>-FAB<br>-RAVLT ir and dr<br>-SCWT<br>-TMT-A/B<br>-Phonemic, Verbal Fluency (COWAT)<br>-RCPM<br>-CDT<br>-Copy of figures<br>-MAC-Q<br>-VRT	**Emotional outcomes:**<br>-HRS-D<br>-TAS<br>**Social outcomes:**<br>-EQ-short<br>-Faux pas Test - Adult Version	**Cognitive outcomes:**<br>Both EG1 and EG2 groups:<br>↑ in subjective perception of memory performance, MoCA and FAB scores *vs.* aCG group<br>EG2 group:<br>↑ MoCA and FAB scores *vs.* aCG group<br>aCG group:<br>↑ executive-attentive and visuospatial domains<br>**Socio-emotional outcomes:**<br>Both EG1 and EG2 groups:<br>↑ mood and TOM *vs.* aCG group

Source	Study Design	Experimental Treatment	Control Treatment	Main Outcome	Secondary Outcomes	Results
		2.Tele-VR cognitive and socio-cognitive training (EG2)<br>**Duration:** 6 weeks<br>**Intensity:** 3 sessions a week<br>**Type of treatment:** remote program via one cognitive rehabilitation app, and one social-cognitive rehabilitation app; in which the patient overcame social challenges with audiovisual feedback.	and including various types of exercises were used.			
**Gobbi et al., 2021**	Randomised controlled trials with crossover features	1.Multimodal training<br>**Duration:** 32 weeks<br>**Intensity:** twice a week<br>**Type of treatment:** trainings for improving/ maintaining all components of functional capacity.<br>Individuals enrolled in the Multimodal exercise group in the first year were switched to the Functional Mobility or Mental/Leisure group in the second year, and Mental/Leisure or Functional Mobility for the third year.	Mental/Leisure training<br>**Duration:** 32 weeks<br>**Intensity:** twice a week<br>**Type of treatment:** cognitive and leisure activities. This program included 2 periods, including 3 sub-periods each. The sub-periods, based on different leisure dimensions (social, manual, and artistic), were always combined with intellectual and social aspects, such as social activities, math problem-solving, card and memory games, drawing, debates, and lectures.	**Cognitive outcomes:**<br>-MMSE<br>-CDT<br>-WMS-R<br>-WAIS-III (Digital span, Search symbol)<br>-WCST<br>-Verbal Fluency test<br>-CBTT	**Psychological outcomes:**<br>-HADS<br>-LSSI<br>-PDQ-39<br>**LPA:**<br>Modified Baecke Questionnaire for Older Adults	**Cognitive outcomes:**<br><u>Multimodal training group:</u><br>•↑ executive function, attention, and working memory *vs.* Functional Mobility training and Mental/Leisure training groups<br>•not have any substantial benefits on executive functions at the 8 months follow-up<br>•not able to delay the progressive decline in cognitive functions at the 8 months follow-up<br>**Emotional outcomes:**<br><u>Multimodal training group:</u><br>↓ physical stress

Source	Study Design	Experimental Treatment	Control Treatment	Main Outcome	Secondary Outcomes	Results
		2.Functional Mobility training<br>**Duration:** 32 weeks<br>**Intensity:** twice a week<br>**Type of treatment:** trainings for improve/maintain balance and locomotion parameters as well as functional capacity and participants' QoL.				
**Bernini et al., 2021**	3 arm double-blind randomised controlled trials	CoRe cognitive training (CCT)<br>**Duration:** 3 weeks<br>**Intensity:** 4 sessions a week<br>**Type of treatment:** CoRe, a software tool, administered 11 tasks targeting several cognitive abilities. These tasks were computerized versions of existing paper-and-pencil exercises or were created to meet specific requirements.<br>The individual patient's performance was analysed to set the appropriate difficulty level which progressively increased.	1.Paper-pencil cognitive training (PCT)<br>**Duration:** 3 weeks<br>**Intensity:** 4 sessions a week<br>**Type of treatment:** same training program as the CCT group but using the paper-and-pencil version of the tasks. The increasing levels of difficulty were managed by the therapist.<br>2.Unstructured activity training (CG)<br>**Duration:** 3 weeks<br>**Intensity:** 4 sessions a week<br>**Type of treatment:** unstructured activities that served as a behavioural placebo treatment.	**Global cognitive functioning:** MoCA	**Episodic long-term memory:**<br>-Logical Memory Test ir and dr<br>-RAVLT ir and dr<br>-ROCFT-dr<br>**Logical-executive functions:**<br>-RCPM<br>-FAB<br>-Semantic, phonological fluency<br>-ROCFT-copy<br>**Working memory:**<br>-Verbal Span<br>-Digit Span<br>-CBTT<br>**Attention/ processing speed:**<br>-Attentive Matrices<br>-TMT-A and TMT-B<br>**Global cognitive functioning:** MMSE	**Cognitive outcomes:**<br><u>CCT group:</u><br>↑ MoCA scores, attention, and processing speed domains *vs.* PCT and CG groups<br><u>PCT group:</u><br>↑ attention/processing speed domain *vs* CG group

Source	Study Design	Experimental Treatment	Control Treatment	Main Outcome	Secondary Outcomes	Results
**Mariano Barboza et al., 2019**	Randomised clinical trial	Cognitive-Motor training (CMG)<br>**Duration:** 16 weeks<br>**Intensity:** twice a week<br>**Type of treatment:** intervention performed in two parts: the same protocol used in the MG and, at the end of each therapy session, 30 minutes of cognitive paper-pencil tasks with gradually increased difficulty. The participants received three more activities to perform at home, which were reviewed in the next session.	Motor training (MG)<br>**Duration:** 16 weeks<br>**Intensity:** twice a week<br>**Type of treatment:** protocol focused on balance training, sensory integration, agility and motor coordination, exploration of limits of stability, anticipatory and reactive postural adjustments, functional independence, and gait improvement. The therapy sessions were divided into four blocks with a gradual increase in exercise complexity.	**Cognitive functions:**<br>-MMSE<br>-MoCA<br>-Semantic Verbal Fluency Test<br>-RAVLT<br>-Cognitive and Perceptual Assessment<br>-TMT<br>-CDT	**Mood and QoL:**<br>-PDQ-39<br>-GDS<br>**Disease severity:**<br>UPDRS	**Cognitive outcomes:**<br><u>Both CMG and MG groups:</u><br>↑ short-term memory and visuospatial function
**Alloni et al., 2018**	Single-blind randomised controlled trials	CoRe cognitive training (G1)<br>**Duration:** 4 weeks<br>**Intensity:** 3 sessions a week<br>**Type of treatment:** CoRe system (Cognitive Rehabilitation); a software tool that automatically generates patient-tailored exercises using a big set of stimuli organised into an ontology.	Sham training (G2)<br>**Duration:** 4 weeks<br>**Intensity:** 3 sessions a week<br>**Type of treatment:** only sham intervention; no cognitive training.	**Global cognitive function:**<br>-MMSE<br>-MOCA<br>**Logical-executive functions:**<br>-RCPM<br>-WEIGL<br>-FAB<br>-Semantic and phonological fluency	**Verbal and spatial memory:**<br>-Verbal Span<br>-Digit Span<br>-CBTT<br>-Logical Memory Test, ir and dr<br>-RAVLT ir and dr<br>-ROCF-dr<br>**Attention:**<br>-Attentive Matrices<br>-TMT-A and TMT-B<br>-SCWT<br>**Visuospatial abilities:**<br>ROCF-copy	**Cognitive outcomes:**<br><u>G1 group:</u><br>↑ executive and memory functions vs. G2 (not maintained after the discharge)

Source	Study Design	Experimental Treatment	Control Treatment	Main Outcome	Secondary Outcomes	Results
**Lawrence et al., 2018**	Randomised controlled trials	1.Tailored cognitive training<br>**Duration:** 4 weeks<br>**Intensity:** 3 sessions a week<br>**Type of treatment:** patients completed individualized activities on Smartbrain Pro; an interactive computer-based training program designed to train each cognitive domain. Performance was automatically monitored by the program to adjust individual difficulty levels for each activity.<br>2.Tailored cognitive training + tDCS<br>**Duration:** 4 weeks<br>**Intensity:** 3 sessions a week + tDCS once a week<br>**Type of treatment:** same as tailored cognitive training, plus 20 minutes of tDCS.	1.Standard cognitive training<br>**Duration:** 4 weeks<br>**Intensity:** 3 sessions a week<br>**Type of treatment:** computer-based training. Predetermined program comprising 10 activities, two activities per cognitive domain<br>2.Standard cognitive training + tDCS<br>**Duration:** 4 weeks<br>**Intensity:** 3 sessions a week + tDCS once a week<br>**Type of treatment:** same as standard cognitive training, plus 20 minutes of stimulation.<br>3.Only tDCS<br>**Duration:** 4 weeks<br>**Intensity:** once a week<br>**Type of treatment:** 20 minutes of stimulation.<br>4.Waiting list<br>**Duration:** N.A.<br>**Intensity:** N.A.<br>**Type of treatment:** participants completed baseline, post-intervention, and 12-week follow-up neuropsychological assessments but did not complete cognitive training or tDCS.	**Executive function:**<br>-SOC (CANTAB)<br>-COWAT<br>**Attention and working memory:**<br>-LNS<br>-SCWT<br>**Memory:**<br>-HVLT-R ir<br>-Paragraph Recall test<br><br>**Visuospatial abilities:**<br>-JLO test<br>-HVOT<br><br>**Language:**<br>-BNT<br>-Similarities test<br><br>**Global cognition:**<br>-PD-CRS<br>-MMSE	**ADL:**<br>-UPDRS-II<br>**QoL:**<br>-PDQ-39	**Cognitive outcomes:**<br><u>Standard cognitive training group:</u><br>↑ memory<br><u>Tailored cognitive training group:</u><br>↑ attention and working memory<br><u>tDCS group:</u><br>↑ attention, working memory and memory<br><u>Standard cognitive training+tDCS group:</u><br>↑ executive function, attention and working memory<br><u>Tailored cognitive training+tDCS group:</u><br>↑ executive function, attention/working memory, and memory<br>**Autonomy and QoL outcomes:**<br><u>Standard cognitive training group:</u><br>↑ADL and QoL<br><u>Tailored cognitive training group:</u><br>↑QoL<br><u>Standard cognitive training+tDCS group:</u><br>↑ ADL
**Kalbe et al., 2020**	Multicentre randomised	NEUROvitalis Parkinson training (CT)	Low-intensity physical activity training (CG)	**Memory:**	**Attention:**	**Cognitive outcomes:**<br><u>CT group:</u>

Source	Study Design	Experimental Treatment	Control Treatment	Main Outcome	Secondary Outcomes	Results
	controlled trials	**Duration:** 6 weeks<br>**Intensity:** twice a week<br>**Type of treatment:** intervention targeting executive functions, memory, attention, and visuo-cognition. Each session is characterized by several training elements: psychoeducation group tasks and activity games, individual exercises, and homework.	**Duration:** 6 weeks<br>**Intensity:** twice a week<br>**Type of treatment:** intervention aimed to be beneficial for PD patients but to have minimal effects on cognition. The main trained domains are stretching, flexibility, loosening up, and relaxation; also, psychoeducation on PD symptoms and therapy options and homework was conducted.	-CVLT total score trials 1-5<br>-CVLT-II dr<br>-ROCFT-dr<br>**Executive functions:**<br>-Semantic, phonemic word fluency<br>-MCST categories<br>-Key search raw score	d2-R concentration performance, errors<br>**Working memory:**<br>WAIS (Letter-number sequencing, Digit span backward)<br>**Visuocognition:**<br>-ROCFT-copy<br>-Benton judgment of line orientation<br>**Language:**<br>-BNT<br>-ACL<br>**Clinical evaluation:**<br>-ADL<br>-Self-reported physical activity<br>-depression<br>-QoL<br>-self-experienced attention deficits<br>-UPDRS-III<br>-FoG-Q	↑ executive functions (especially verbal fluency), but not memory<br><u>CG group:</u><br>↑ working memory
**Reuter et al., 2012**	Blind randomised study	Cognitive, transfer and psychomotor training (Group C):<br>**Duration:** 3-4 weeks + prosecution at home<br>**Intensity:** 4 cognitive training sessions a week + 3 transfer training sessions a week + 3 psychomotor training sessions a week + at home 3 cognitive training	1.Transfer and cognitive training (Group B)<br>**Duration:** 3-4 weeks + prosecution at home<br>**Intensity:** 4 cognitive training sessions a week + 3 transfer training sessions a week + at home 3 cognitive training sessions a week, 2 transfer training sessions a week, and 2 relaxation training a week.	**Alzheimer Cognition:**<br>ADAS-COG subscale	**Parkinson's disease Cognition:**<br>SCOPA-COG	**Cognitive outcomes:**<br><u>Group C:</u><br>↑ cognitive performance *vs.* Group B and A<br><u>Group B:</u><br>↑ cognitive performance *vs.* Group A

Source	Study Design	Experimental Treatment	Control Treatment	Main Outcome	Secondary Outcomes	Results
		sessions a week, 2 transfer training sessions a week and 2 psychomotor training sessions a week.<br>**Type of treatment:** cognitive training in addition to transfer and psychomotor training, with a prosecution at home. The cognitive training employed a computer-based programme, and it included training of different cognitive functions. For transfer training, patients were asked to practise competence in tasks of daily routines. Psychomotor training included games and tasks designed to learn how to perform motor sequences. Also, mental imagery and aerobic training were employed.	**Type of treatment:** same cognitive and transfer training as Group C, with a prosecution at home. While at home, instead of the same psychomotor training of Group C, participants performed relaxation training.<br>2.Only cognitive training (Group A)<br>**Duration:** 3-4 weeks + prosecution at home<br>**Intensity:** 4 cognitive training sessions a week + at home 3 cognitive training sessions a week, 2 transfer training sessions a week, and 2 relaxation training a week.<br>**Type of treatment:** same cognitive training of Group C and B, with a prosecution at home.			
**Prat Paris et al., 2011**	Blind multicentre randomised controlled trials	Cognitive training (CTG)<br>**Duration:** 4 weeks<br>**Intensity:** 3 sessions a week<br>**Type of treatment:** interactive multimedia software	Speech therapy<br>**Duration:** 4 weeks<br>**Intensity:** 3 times a week group sessions + once a week individual tutored session<br>**Type of treatment:**	**Cognitive performance:**<br>-MMSE<br>-ACE-III<br>**Attention and working memory:**	**Depression:**<br>GDS-15<br>**QoL:**<br>PDQ- 39<br>**Cognitive difficulties in ADLs:**<br>CDS	**Cognitive outcomes:**<br><u>CTG group:</u><br>↑ in attention, information processing speed, memory, visuospatial and visuoconstructive abilities, semantic verbal fluency, and

Source	Study Design	Experimental Treatment	Control Treatment	Main Outcome	Secondary Outcomes	Results
		and paper-and-pencil exercises. Computer-aided training employed the SmartBrain tool, designed to stimulate specific and non-specific cognitive domains. Participants received a pack with 20 cognitive homework exercises designed to stimulate specific and nonspecific cognitive areas.	speech therapy aimed to make participants aware of their speech and communication difficulties.	-Digits subtest (WAIS-III)<br>-CVLT-II-List A1<br>**Information processing speed:**<br>-SDMT<br>-TMT-A<br>-SCWT<br>**Verbal memory:**<br>-CVLT-II<br>-Logical Memory subtest (WMS-III)<br>**Learning:**<br>CVLT-II<br>**Visual memory and visuo-constructive abilities:**<br>ROCFT<br>**Visual spatial:**<br>RBANS<br>**Verbal fluency:**<br>-FAS<br>-semantic, using animals<br>**Frontal lobe sensitivity:**<br>-TOL<br>-TMT-B<br>-SCWT		executive functions *vs.* Speech therapy group<br>**Autonomy and QoL outcomes:**<br><u>CTG group:</u><br>no significant improvements in QoL
**Sarasso et al., 2021**	Randomised clinical/ fMRI study	DUAL-TASK+AOT-MI training<br>**Duration:** 6 weeks<br>**Intensity:** 3 sessions a week<br>**Type of treatment:**	DUAL- TASK training<br>**Duration:** 6 weeks<br>**Intensity:** 3 sessions a week<br>**Type of treatment:**<br>patients performed the same number of exercises combined	**Cognitive dual task:**<br>TUG-COG	**Turning velocity parameters** during the execution of:<br>-TUG<br>-TUG-COG<br>-TUG-MAN	**Motor outcomes:**<br><u>Both groups:</u><br>↑ in mobility during TUG-COG, TUG-MAN, and TUG (maintained 2 months after training)

Source	Study Design	Experimental Treatment	Control Treatment	Main Outcome	Secondary Outcomes	Results
		patients performed a gait/ balance training consisting of AOT-MI combined with practising the observed-imagined exercises	with watching landscape videos instead of observation/imagination, and exercises were increasingly difficult up to, including the dual task.		**DTC**<br>**Executive functions:**<br>AST subtest (CANTAB)	DUAL-TASK+AOT-MI group:<br>↑ change in TUG-COG mean, and peak of turning velocity during TUG and TUG-COG (maintained at follow-up) *vs.* DUAL-TASK group
**Agosta et al., 2017**	Prospective randomised study	AOT Group<br>**Duration:** 4 weeks<br>**Intensity:** 3 sessions a week<br>**Type of treatment:** physical therapy training: during each training session, two video clips showing strategies useful in circumventing FoG episodes, were presented twice. Overall, subjects in the AOT group were presented with six video clips, repeated each week. The complexity of actions increased, and auditory cues were associated with the movements. After each video clip observation, patients were asked to imitate the observed actions repetitively and accurately at the beat of the auditory cues.	Landscape Group<br>**Duration:** 4 weeks<br>**Intensity:** 3 sessions a week<br>**Type of treatment:** physical therapy training, during each training session they watched video clips containing sequences of static pictures of landscapes without any living representations for the same time length. During training sessions, patients performed the same movements/actions used for the AOT group in the same order and amount of time, following the physical therapist's instructions.	**Clinical and functional state:**<br>-UPDRS-III<br>-H&Y scale<br>-FoG-Q<br>-UPDRS-II FoG score<br>-PDQ-39<br>**Motor functioning:**<br>-BBS<br>-10 M-WT<br>**fMRI and fMRI task**	**Neuropsychological functioning:**<br>-MMSE<br>-RAVLT<br>-ROFCT-copy and dr<br>-Phonemic and Semantic Fluency<br>-CDT<br>-Modified Card Sorting Test<br>-Digit span backward<br>-Attentive matrices<br>-TMT<br>-Naming subtests (BADA)<br>-Token Test<br>-Visual spatial subtests (ACE-R-VS)<br>**Mood:**<br>BDI	**Motor outcomes:**<br>Both groups:<br>•↓ FoG severity<br>•↑ walking speed<br>AOT group:<br>•↓ motor impairment (after short-term follow-up) *vs.* Landscape group<br>•↑ walking speed and balance (after short-term follow-up) *vs.* Landscape group<br>**Autonomy and QoL outcomes:**<br>Both groups:<br>↑ quality of life<br>AOT group:<br>↑ quality of life *vs.* Landscape group (after short-term follow-up)
**Suarez-Garcia et al., 2021**	Randomised blinded sham-	PD-atDCS Group<br>**Duration:** 5 consecutive days	PD-stDCS Group	**Action-verb processing and**	**Cognitive status:**<br>ACE-R<br>**Executive skills:**	**Cognitive outcomes:**<br>PD-atDCS group:

Source	Study Design	Experimental Treatment	Control Treatment	Main Outcome	Secondary Outcomes	Results
	controlled study	**Intensity:** 3 phases<br>**Type of treatment:**<br>3 phases of protocol with stimulation; pre-stimulation phase, stimulation phase, and post-stimulation phase. Both before and after the stimulation protocol, participants completed a PWA task involving action-verb and object-noun conditions. During the stimulation phase, participants received 20 min of online stimulation while completing a cognitive training protocol. The post-stimulation phase was identical in structure and duration to the pre-stimulation phase.	**Duration:** 5 consecutive days<br>**Intensity:** 3 phases<br>**Type of treatment:**<br>same 3 phases of the experimental treatment; except for receiving a sham stimulation, lasting 1 minute and not effective.	**object-noun processing:**<br>RT and trial accuracy during the different tasks	IFS battery<br>**Motor skills:**<br>UPDRS-III	•↑ for action-verb processing *vs.* PD-stDCS group<br>•no effect for object-noun processing
**Vriend et al., 2021**	Double-blind randomised controlled trials	Computerised cognitive training<br>**Duration:** 8 weeks<br>**Intensity:** 3 sessions a week<br>**Type of treatment:**<br>home-based computer-intervention employed 13 training games with adaptive difficulty that focused on different cognitive functions and	Computerised active control group<br>**Duration:** 8 weeks<br>**Intensity:** 3 sessions a week<br>**Type of treatment:**<br>home-based, computer intervention that employed three low-threshold games with constant difficulty primarily based on "crystallized intelligence" factors, i.e.,	**Topology of the structural brain network**	**Executive functions:**<br>self-paced computer-based version of ToL<br>**Processing speed:**<br>SCWT	**Cognitive outcomes:**<br><u>Computerised cognitive training group:</u><br>faster responses on the ToL task *vs.* Computerised active control group<br>**Neurophysiological outcomes:**<br><u>Computerised cognitive training group:</u><br>no effect on network topology neither on the global or subnetwork level

Source	Study Design	Experimental Treatment	Control Treatment	Main Outcome	Secondary Outcomes	Results
		were adapted from the Braingymmer online platform.	solitaire, hangman, and trivia questions.			

Abbreviations in alphabetical order: ↑: increasing in the outcome; ↓: decreasing in the outcome; **10 M-WT:** 10 Metre Walk Test; **ACE-III:** Addenbrooke Cognitive Examination-III; **ACE-R AO:** Addenbrooke Cognitive Examination-Revised Attention and Orientation; **ACE-R F:** Addenbrooke Cognitive Examination-Revised Fluency; **ACE-R L:** Addenbrooke Cognitive Examination-Revised Language; **ACE-R M:** Addenbrooke Cognitive Examination-Revised Memory; **ACE-R VS:** Addenbrooke Cognitive Examination-Revised Visual-Spatial cognition; **aCG:** active Control Group; **ACL:** Aphasia Check List; **ADAS-COG:** Alzheimer Assessment Scale-Cognition; **ADL:** Activities of Daily Living; **AKT:** Alters-Konzentrations Test; **AOT:** Action Observation Training; **AOT-MI:** Action Observation Training-Motor Imagery; **atDCS:** anodal transcranial Direct Current Stimulation; **BADA:** Battery for the assessment of aphasic disorders; **BADS:** Battery of behavioural assessment of the Dysexecutive Syndrome; **BBS:** Berg Balance Scale; **BDI:** Beck Depression Inventory; **BDI-II:** Beck Depression Inventory-II; **Bf-S:** "self-rated mood" scale; **BNT:** Boston Naming Test; **CACR:** computer-assisted cognitive rehabilitation; **CANTAB:** Cambridge Neuro-psychological Test Automated Battery; **CANTAB AST:** Cambridge Neuro-psychological Test Automated Battery Attention Switching Task; **CANTAB MOT:** Cambridge Neuro-psychological Test Automated Battery Motor Screening Test; **CANTAB OTS:** Cambridge Neuro-psychological Test Automated Battery One Touch Stockings of Cambridge; **CANTAB SOC:** Cambridge Neuro-psychological Test Automated Battery-Stockings of Cambridge; **CANTAB SRM:** Cambridge Neuro-psychological Test Automated Battery Spatial Recognition Memory; **CANTAB SWM:** Cambridge Neuro-psychological Test Automated Battery Spatial Working Memory; **CBTT:** Corsi's block-tapping test; **CCT:** Computer-based cognitive training; **CDS:** Cognitive Difficulties Scale; **CDT:** Clock drawing test; **CEQ:** Credibility and Expectancy Questionnaire; **CES-D short form:** Centre for Epidemiologic Studies Short Depression Scale; **CFQ:** Cognitive Failures Questionnaire; **CG:** Control Group; **CMG:** Cognitive motor group; **CoRE:** Cognitive Rehabilitation computer software; **COWAT:** Controlled Oral Word Association Test; **CT:** Cognitive training; **CTG:** cognitive training group; **CVLT:** California Verbal Learning Test; **CVLT-II:** California Verbal Learning Test-II; **CVLT-II-List A1:** California Verbal Learning Test-II-List A1; **d2-R:** d2 Test of Attention-Revised; **dr:** delayed recall; **DTC:** Dual-task cost; **EF:** executive functions; **EG1:** Experimental Group 1; **EG2:** Experimental Group 2; **EQ-short:** The Short Empathy Quotient Scale; **ERICA:** Esercizi di Riabilitazione Cognitiva (Exercises of Cognitive Rehabilitation); **FAB:** Frontal Assessment Battery; **FAS:** phonemic word fluency test; **fMRI:** functional Magnetic Resonance Imaging; **FoG:** Freezing of Gait; **FoG-Q:** Freezing of Gait-Questionnaire; **G1:** Group 1; **G2:** Group 2; **GDS:** Geriatric Depression Scale; **GDS-15:** Geriatric Depression Scale-15; **GNL:** Face-Name-Learning Test; **H&Y** scale: Hoehn and Yahr Scale; **HAMD:** Hamilton Depression Rating Scale; **HRS-A:** Hamilton Rating Scale Anxiety; **HRS-D:** Hamilton Rating Scale for Depression/Anxiety; **HVLT-R ir:** Hopkins Verbal Learning Test Revised-immediate recall; **HVOT:** Hooper Visual Organisation Test; **IFS battery:** INECO Frontal Screening (IFS) battery; **II:** Implementation Intention; **ir:** immediate recall; **JLO test:** Judgement of Line Orientation; **KVIQ:** Kinaesthetic and Visual Imagery Questionnaire; **LPA:** level of physical activity; **LNS:** Letter-Number Sequencing; **LSSI:** Lipp's Stress Symptoms Inventory; **MAC-Q:** Memory Assessment Clinics-questionnaire; **MCST** categories: Modified Card Sorting Test; **MG:** Motor group; **MI:** Motor Imagery; **MMSE:** Mini Mental State Examination; **MoCA:** Montreal Cognitive Assessment; **MWT:** German test for the assessment of verbal intelligence; **N.A.:** not applicable; **NPI-Q:** Neuropsychiatric Inventory Questionnaire; **NPI-Q14:** Neuropsychiatric Inventory Questionnaire-14; **PCT:** Paper-pencil cognitive training; **PD:** Parkinson's disease; **PD-atDCS Group:** Parkinson's disease-anodal transcranial Direct Current Stimulation Group; **PD-CFRS:** Parkinson's disease Cognitive Functional Rating Scale; **PD-CFRS inf:** Parkinson's disease Cognitive Functional Rating Scale informant version; **PD-CRS:** Parkinson's disease-Cognitive Rating Scale; **PD-MCI:** Parkinson's disease-Mild Cognitive Impairment; **PDQ:** Parkinson's disease

Questionnaire; **PDQ-39:** Parkinson's disease Questionnaire-39; **PD-stDCS Group:** Parkinson's disease-sham transcranial Direct Current Stimulation Group; **PKMAS:** ProtoKinetics Movement Analysis Software; **PRMQ-Pro:** Prospective and Retrospective Memory Questionnaire-Pro; **PRMQ subscale Pro-Env:** Prospective and Retrospective Memory Questionnaire subscale environment-cued; **PRMQ subscale Pro-Self:** Prospective and Retrospective Memory Questionnaire subscale self-cued; **PT:** Physical training; **PWA:** picture-word association; **QoL:** quality of life; **RAVLT:** Rey Auditory Verbal Learning test; **RAVLT-dr:** Rey Auditory Verbal Learning test-delayed recall; **RAVLT-ir:** Rey Auditory Verbal Learning test-immediate recall; **RBANS:** Repeatable Battery for the Assessment of Neuropsychological Status; **RCPM:** Raven's Coloured Progressive Matrices; **REHACOP:** cognitive rehabilitation program in psychosis; **ReSET training:** Strategic Executive Treatment; **RM47:** Raven's Matrices; **ROCFT-dr:** Rey-Osterrieth Complex Figure Test-delayed recall; **ROCFT-ir:** Rey-Osterrieth Complex Figure Test-immediate recall; **RRL:** The Role Resumption list; **RS-FC:** Resting-state functional connectivity; **RT:** response time; **SCOPA-COG:** Scales for outcome of Parkinson's Disease-Cognition; **SCT:** standard cognitive training; **SCWT:** Stroop Colour and Word Test; **SDMT:** Symbol-Digit Modalities Test; Short battery **ACE-III:** Short battery Addenbrooke Cognitive Examination-III; **SOPT:** speed of processing training; **stDCS:** sham transcranial Direct Current Stimulation; **TAS:** Toronto Alexithymia Scale; **tDCS:** transcranial Direct Current Stimulation; **TEA:** Test of Everyday Attention; **TEE:** total energy expenditure; **TGA:** Treatment goal attainment; TKS: Cognitive Estimation Test; **TMT:** Trail Making Test; **TMT-A:** Trail Making Test-Part A; **TMT-B:** Trail Making Test-Part B; **TMT-B/A:** Trail Making Test- Part B/A; **ToL:** Tower of London; **TOM:** theory of mind; **TT:** Treadmill training; **TT+VR:** Treadmill training + Virtual Reality; **TUG:** Timed-Up-and–Go test; **TUG-COG:** Timed-Up-and–Go test-Cognitive; **TUG-MAN:** Timed-Up-and–Go test-Manual task; **UFOV:** Useful Field of View Test; **UPDRS:** Unified Parkinson's disease Rating Scale; **UPDRS-II:** Unified Parkinson's disease Rating Scale-II; **UPDRS-II FoG:** Unified Parkinson's disease Rating Scale-II Freezing of Gait; **UPDRS-III:** Unified Parkinson's disease Rating Scale-III; **VR:** Verbal Rehearsal; **VRT:** Verbal Reasoning Assessment Test; **WAIS:** Wechsler Adult Intelligence Scale; **WAIS-III:** Wechsler Adult Intelligence Scale-III; **WCST:** Wisconsin Card Sorting Test; **WEIGL:** Weigl's Sorting Test; **WHO-DAS II:** World Health Organization-Disability Assessment Schedule II; **WMS-R:** Wechsler Memory Scale-revised; **ZVT:** Zahlen-Verbindungs-Test (Trial Making Test German version).
