# Supplementary material for: Identification of Cognitive Training for Individuals with Parkinson’s Disease: A Systematic Review
Source: Brain Sci. 2025 Jan 11;15(1):61. doi: 10.3390/brainsci15010061 (PMC11763612; doi:10.3390/brainsci15010061)
Supplement: Supplementary file 1 [file brainsci-15-00061-s001.zip › Table S2.Studies and sample characteristics.pdf]

Supplementary Materials – Table S2. Studies and sample characteristics

ID	Source	Country	Inclusion Criteria	Exclusion Criteria	Sample (N, Gender, Age)	PD Stage (M, SD/N, %)
1	**Bode et al., 2023**	Germany	-Diagnosis of PD (UK Parkinson's Disease Brain Bank Criteria)<br>-Age: 50-80 years<br>-Presence of self-reported cognitive impairment (SCI questionnaire) and/or MoCA<26<br>-Presence of PD-MCI (MDS Task Force Level-II criteria)<br>-PD duration: ≥3 years<br>(Taken from Kalbe et al., 2020)	-BDI-II ≥20<br>-Impaired ADL<br>-Acute suicide tendency<br>-Severe comorbidities affecting life expectancy, medication, or QoL<br>-Severe fatigue<br>-Prominent impulse control disorder or dopamine dysregulation syndrome<br>-Acute psychosis or psychotic episode in the last 6 months<br>-Dementia medication<br>-Participation in other treatment studies within the last 2 months<br>-DBS<br>-Pregnancy or nursing period<br>(Taken from Kalbe et al., 2020)	**NEUROvitalis training:** n=8, 6 males (75.0%), 66.00 (7.93)<br>**Physical training:** n=10, 5 males (50.0%), 70.70 (9.18)	**NEUROvitalis training:**<br>-H&Y stage: range 2-3<br>-UPDRS-III: 22.25 (8.38)<br>**Physical training:**<br>-H&Y stage: range 1-3<br>-UPDRS-III: 28.20 (10.04)
2	**van Balkom et al., 2022**	Netherlands	-Diagnosis of PD (neurological evaluation)<br>-H&Y stage <4<br>-PD-CFRS >3	-MoCA <22<br>-CAGE-AID-interview >1<br>-BDI >18<br>-Impulse control disorder<br>-Non-benign psychotic symptoms<br>-History of traumatic brain injury with loss of consciousness	**Computerised cognitive training:** n=68, 35 males (51%), 62.9 (8.1)<br>**Active control:** n=68, 47 males (69%), 62.9 (7.0)	**Computerised cognitive training:**<br>-H&Y stage: range 1-3<br>-UPDRS-III: 20.2 (8.3)<br>**Active control:**<br>-H&Y stage: range 1-3<br>-UPDRS-III: 21.0 (9.5)
3	**Sousa et al., 2021**	Brazil	-Diagnosis of PD<br>-Presence of PD-MCI (MDS Task Force Level-II criteria)<br>-H&Y stages 1-3	Not specified	**Paper-pencil cognitive training:** n=24, 20 males (83.33%), 60.0 (7.5)<br>**General rehabilitation:** n=15, 13 males (86.66%), 58.5 (9.8)	**Paper-pencil cognitive training:**<br>-H&Y stage: range 1-3<br>**General rehabilitation:**<br>-H&Y stage: range 1-3

ID	Source	Country	Inclusion Criteria	Exclusion Criteria	Sample (N, Gender, Age)	PD Stage (M, SD/N, %)
4	**Vlagsma et al., 2020**	Netherlands	-Diagnosed of PD (UK Parkinson's Disease Brain Bank Criteria)<br>-Age:18-80 years<br>-H&Y stage ≤3<br>-Presence of problems with EF in everyday life (semi-structured interview and/or Dysexecutive Questionnaire ≥18)<br>-Presence of impairments on objective neuropsychological tests of EF	Severe neurological and psychiatric comorbidity including dementia	**ReSET training:** n=24, 14 males (58%), 60.21 (10.42)<br>**CogniPlus training:** n=19, 13 males (68%), 62.58 (8.84)	**ReSET training:**<br>-UPDRS-III: 23.96 (9.46)<br>-H&Y stage: 2.37 (0.57)<br>**CogniPlus training:**<br>-UPDRS-III: 22.24 (11.44)<br>-H&Y stage: 2.19 (0.39)
5	**De Luca et al., 2019**	Italy	-Diagnosis of PD (MDS Clinical Diagnostic Criteria for Parkinson's disease)<br>-H&Y stage <3<br>-Presence of mild-to moderate cognitive impairment (MoCA 18-24)	-Age: >85 years<br>-Presence of severe medical and psychiatric illness potentially interfering with the training	**Computerised cognitive training:** n=30, 16 males (53.3%), 61.9 (11.5)<br>**Standard cognitive training:** n=30, 15 males (50.0%), 63.2 (7.3)	**Total sample:**<br>H&Y stage: range 1.0-2.5
6	**Bernini et al., 2019**	Italy	-Diagnosis of PD (UK Parkinson's Disease Brain Bank Criteria)<br>-Age: 50-85 years<br>-H&Y stage ≤4<br>-Presence of single-domain (executive) or multiple-domain PD-MCI with executive involvement	-Pre-existing cognitive impairment<br>-Severe disturbances in consciousness<br>-Severe sensory or motor disturbances; patients with disturbing resting and/or action<br>-DBS<br>-Concomitant severe psychiatric or neurological conditions	**CoRe cognitive training + standard physical training:** n=17, 6 males (35.29%), 71.18 (7.04)<br>**Standard physical training:** n=18, 11 males (61.11%), 69.33 (7.72)	**CoRe cognitive training + standard physical training:**<br>-H&Y stage: 2.8 (0.96)<br>-UPDRS-III: 37.82 (13.93)<br>**Standard physical training:**<br>-H&Y stage: 2.9 (0.47)<br>-UPDRS-III: 36.50 (12.82)
7	**Goedeken et al., 2018**	United States of America	-Diagnosis of PD (UK Parkinson's Disease Brain Bank Criteria)<br>-Age: ≥50 years<br>-H&Y stages 1-3<br>-Possible presence of PD-MCI	-Suspected dementia or global cognitive impairment determined by MDS or MMSE <27<br>-Currently taking medications that interfere with cognitive function<br>-Change in medication over the course of the study	**Implementation intentions training:** n=25, 12 males (48%), 63.8 (4.6)<br>**Verbal rehearsal training:** n=27, 13 males (48.15%), 62.7 (5.5)	**Implementation intentions training:**<br>-H&Y stage: range 1-3<br>-UPDRS: 17.2 (10.0)<br>**Verbal rehearsal training:**<br>-H&Y stage: range 1-3<br>-UPDRS: 15.3 (6.9)

ID	Source	Country	Inclusion Criteria	Exclusion Criteria	Sample (N, Gender, Age)	PD Stage (M, SD/N, %)
				-Other neurological disorders<br>-History of brain surgery<br>-History of or current psychotic disorder<br>-Current psychiatric conditions that could interfere with study participation		
**8**	**Peña et al., 2014**	Spain	-Diagnosis of PD<br>-Age: 45-75 years<br>-H&Y stages 1-3	-Presence of dementia (DSM-IV-TR11, MDS)<br>-Presence of other neurologic illness or injury<br>-Unstable psychiatric disorders such as schizophrenia or major depression<br>-Presence of visual hallucinations (NPI-Q)	**REHACOP cognitive training:** n=22, 13 males (59.1%), 67.55 (5.2)<br>**Occupational training:** n=22, 14 males (63.6%), 68.13 (7.5)	**REHACOP cognitive training:**<br>-H&Y stage: range 1-2<br>-UPDRS total score: 33.6 (13.8)<br>**Occupational training:**<br>-H&Y stage: range 1-3<br>-UPDRS total score: 39.0 (20.4)
**9**	**Petrelli et al., 2014**	Germany	Diagnosis of PD (UK Parkinson's Disease Brain Bank Criteria)	-MMSE <25<br>-Other neurological or psychiatric diseases (except for depression)<br>-Impaired hearing or sight, and treatment with<br>-DBS	**NEUROvitalis structured cognitive training:** n=22, 10 males (45.45%), 69.2 (4.9)<br>**Mentally fit unstructured cognitive training:** n=22, 15 males (68.18%), 68.8 (6.7)<br>**Waiting list:** n=21, 12 males (57.14%), 69.1 (11.6)	**NEUROvitalis structured cognitive training:**<br>-UPDRS-III: 21.1 (7.6)<br>**Mentally fit unstructured cognitive training:**<br>-UPDRS-III: 19.7 (8.5)<br>**Waiting list:**<br>-UPDRS-III: 20.9 (6.6)
**10**	**Zimmermann et al., 2014**	Switzerland	Diagnosis of PD (UK Parkinson's Disease Brain Bank Criteria)	-Moderate or severe dementia<br>-Other severe neurologic conditions	**CogniPlus training:** n=19, 13 males (68%), 69.9 (6.3)<br>**Nintendo Wii exergames training:**	**CogniPlus training:**<br>-H&Y stage: 2 (0, 2.5)<br>-UPDRS-Total: 24 (7.5, 33)<br>**Nintendo Wii exergames training:**

ID	Source	Country	Inclusion Criteria	Exclusion Criteria	Sample (N, Gender, Age)	PD Stage (M, SD/N, %)
					n=20, 12 males (60%), 66.3 (9.7)	-H&Y stage: 2 (1.12, 2.5)<br>-UPDRS-Total: 25 (21.75, 34)
**11**	**Sammer et al., 2006**	Germany	-Diagnosis of idiopathic PD<br>-H&Y stages 2-3	Not specified	**Executive function training:** n=12, 70.8 (7.9)<br>**Standard treatment:** n=14, 68.5 (9.0)	**Total sample:**<br>H&Y stage: range 2-3
**12**	**Maidan et al., 2017**	Israel	-Diagnosis of idiopathic PD<br>-Age: 60-90 years<br>-H&Y stages 2-3	-MMSE <24<br>-Contraindications to undergo MRI examination<br>-Psychiatric comorbidity<br>-History of other neurologic disorder<br>-Orthopaedic problems, or unstable medical condition	**Treadmill training + VR:** n=17, 11 males (64.71%), 71.2 (1.7)<br>**Treadmill training:** n=17, 12 males (70.59%), 71.5 (1.5)	**Treadmill training + VR:**<br>-UPDRS-III: 26.9 (3.2)<br>-H&Y stage: range 2-3<br>**Treadmill training:**<br>-UPDRS-III: 30.8 (3.7)<br>-H&Y stage: range 2-3
**13**	**Edwards et al., 2013**	United States of America	-Diagnosis of idiopathic PD,<br>-Age: ≥40 years<br>-H&Y stages 1-3<br>-MMSE ≥ 24	-Unpredictable or severe motor fluctuations<br>-Dyskinesias	**Cognitive speed of processing training:** n=44, 28 males (63.64%), 69.38 (7.81)<br>**Waiting list:** n=43, 26 males (60.47%), 68.17 (8.38)	**Cognitive speed of processing training:**<br>-H&Y stage: range 1-3<br>**Waiting list:**<br>-H&Y stage: range 1-3
**14**	**Pompeu et al., 2012**	Brazil	-Diagnosis of idiopathic PD<br>-Age: 60-85 years<br>-H&Y stages 1-2<br>-No other neurological or orthopaedic diseases<br>-MMSE >23<br>-GDS >6	Not specified	**Total sample:** n=32, 17 males (53.12%), 67.4 (8.1)	**Total sample:**<br>-H&Y stage 1.7 (0.5)
**15**	**Leocadi et al., 2024**	Italy	-Diagnosis of PD PIGD phenotype (MDS-UPDRS ≤ 0.9)<br>-H&Y stage ≤4<br>-Tremor-dominant/PIGD ratio<br>-Possible presence of PD-MCI	-Presence of medical illnesses or substance abuse that could interfere with cognition<br>-Other major systemic, psychiatric, neurological, visual	**DUAL-TASK +AOT-MI training:** n=11, 6 males (54.55%), 68.50 (5.95)	**DUAL-TAK + AOT-MI training:**<br>-H&Y stage: 2.41 (0.43)<br>-UPDRS-III: 28.32 (9.33)

ID	Source	Country	Inclusion Criteria	Exclusion Criteria	Sample (N, Gender, Age)	PD Stage (M, SD/N, %)
			-MMSE ≥24	and musculoskeletal disturbances or other causes of walking inability<br>-Contraindications to undergo MRI examination<br>-Brain damage at routine MRI, including lacunae and extensive cerebrovascular disorders	**DUALT-TASK training:** n=10, 6 males (60%), 63.42 (9.9)	-PD-MCI: 4 patients (36.36%)<br>**DUAL-TASK training:**<br>-H&Y stage: 2.30 (0.35)<br>-UPDRS-III: 20.20 (8.35)<br>-PD-MCI: 3 patients (30%)
**16**	**Maggio et al., 2024**	Italy	-Diagnosis of PD (Movement Disorder Society Clinical)<br>-Age: 40-80 years<br>-H&Y stage <2.5<br>-No cognitive impairment	-Psychiatric disorders<br>-Dementia	**Tele-VR cognitive training:** n=12, 8 males (66.7%), 59.7 (9.7)<br>**Tele-VR cognitive and socio-cognitive training:** n=12, 6 males (50.0%), 63.8 (8.3)<br>**Not-VR cognitive training:** n=10, 9 males (90%), 66.8 (6.5)	**Tele-VR cognitive training:** UPDRS-III: 27.9 (12.7)<br>**Tele-VR cognitive and socio-cognitive training:** UPDRS-III: 22.1 (6.3)<br>**Not-VR cognitive training:** UPDRS-III: 33.8 (8.3)
**17**	**Gobbi et al., 2021**	Brazil	-Diagnosis of PD (UK Parkinson's Disease Brain Bank Criteria)<br>-Age: >40 years<br>-No other neurological disorders<br>-No cognitive impairment	Not specified	**Multimodal training:** n=57, 31 males (54%), 69.6 (8.2)<br>**Functional mobility training:** n=48, 28 males (58%), 67.8 (9.1)<br>**Mental/leisure training:** n=47, 19 males (40%), 69.5 (7.6)	**Multimodal training:**<br>-H&Y stage: 1.9 (0.6)<br>-UPDRS-Total score: 38.3 (12.5)<br>**Functional mobility training:**<br>-H&Y stage: 1.8 (0.6)<br>-UPDRS-Total score: 37.5 (14.2)<br>**Mental/leisure training:**<br>-H&Y stage: 1.7 (0.5)<br>-UPDRS-Total score: 37.7 (13.7)
**18**	**Bernini et al., 2021**	Italy	-Diagnosis of PD (UK Parkinson's Disease Brain Bank Criteria)<br>-Age: 50-85 years<br>-H&Y stage ≤3	-Cognitive impairment due to a pre-existing neurological condition	**CoRe cognitive training:** n=18, 12 males (67%), 74.61 (5.68)	**CoRe cognitive training:**<br>-UPDRS-III: 34.93 (9.31)<br>**Paper-pencil cognitive training:**

ID	Source	Country	Inclusion Criteria	Exclusion Criteria	Sample (N, Gender, Age)	PD Stage (M, SD/N, %)
			-Presence of single or multiple-domain PD-MCI	-Concomitant severe psychiatric disease and/or non-neurological comorbidities<br>-Severe sensory or motor disturbances liable to interfere with the intervention<br>-Deep brain stimulation<br>-Change in medication	**Paper-pencil cognitive training**: n=12, 7 males (58%), 69.83 (9.66)<br>**Unstructured activity training:** n=18, 12 males (67%), 69.33 (7.72)	-UPDRS-III: 30.86 (10.35)<br>**Unstructured activity training:**<br>-UPDRS-III: 36.50 (12.82)
**19**	**Mariano Barboza et al., 2019**	Brazil	-Diagnosis of idiopathic PD (London Brain Bank criteria)<br>-Age: >50 years<br>-H&Y stages 1.5-3	Other neurological, musculoskeletal, and associated disorders and cognitive alterations that could interfere in the evaluation process	**Cognitive-motor training:** n=28, 10 males (35.7%), 67.11 (8.14)<br>**Motor training:** n=26, 12 males (46.2%), 64.33 (7.77)	**Cognitive-motor training:**<br>-H&Y stage: range 1.5-3.0<br>-UPDRS-Total: 33.23 (11.18)<br>**Motor training:**<br>-H&Y stage: range 2-3<br>-UPDRS-Total: 34.18 (12.46)
**20**	**Alloni et al., 2018**	Italy	-Diagnosis of PD (UK Parkinson's Disease Brain Bank Criteria)<br>-Age: 50-85 years<br>-H&Y stage ≤4<br>-Presence of single-domain (executive) or multiple-domain PD-MCI with executive involvement	-Pre-existing cognitive impairment<br>-Severe disturbances in consciousness<br>-Severe psychiatric or neurological conditions<br>-Severe motor or sensory disorders that do not allow control of the trunk and maintenance of the sitting position	**CoRe cognitive training:** n=17, 5 males (29.41%), 71.18 (7.04)<br>**Sham training:** n=14, 9 males (64.29%), 69.53 (8.04)	**Total sample:**<br>H&Y stage: range 1-4
**21**	**Lawrence et al., 2018**	Australia	-Diagnosis of PD (UK Parkinson's Disease Brain Bank Criteria)<br>-Presence of PD-MCI (MDS Level II diagnostic criteria)<br>-UPDRS-II score <3	-Presence of PD-D<br>-Recent history of brain surgery<br>-DBS implant<br>-Active skin disease on the scalp<br>-History of migraine or epilepsy<br>-Metal implants in the head/brain	**Standard cognitive training:** n=7, 3 males (43%), 68.14 (8.69)<br>**Tailored cognitive training:** n=7, 4 males (57%), 65.57 (5.20)	**Standard cognitive training:**<br>UPDRS-II: 0.96 (0.77)<br>**Tailored cognitive training:**<br>UPDRS-II: 0.68 (0.32)<br>**tDCS:**

ID	Source	Country	Inclusion Criteria	Exclusion Criteria	Sample (N, Gender, Age)	PD Stage (M, SD/N, %)
					**tDCS:** n=7, 5 males (71%), 72 (6.45)<br>**Standard cognitive training + tDCS:** n=7, 5 males (71%), 63.57 (15.68)<br>**Tailored cognitive training + tDCS:** n=7, 5 males (71%), 67.43 (6.37)<br>**Waiting list:** n=7, 4 males (57%), 72.29 (6.21)	UPDRS-II: 1.27 (0.56)<br>**Standard cognitive training + tDCS:**<br>UPDRS-II: 1 (0.48)<br>**Tailored cognitive training + tDCS:**<br>UPDRS-II: 1.17 (0.56)<br>**Waiting list:**<br>UPDRS-II: 1.18 (0.69)
**22**	**Kalbe et al., 2020**	Germany	-Diagnosis of PD (UK Parkinson's Disease Brain Bank Criteria)<br>-Age: 50-80 years<br>-Presence of self-reported cognitive impairment (SCI questionnaire) and/or MoCA<26<br>-Presence of PD-MCI (MDS Task Force Level-II criteria)<br>-PD duration: ≥3 years	-BDI-II ≥20<br>-Impaired ADL<br>-Acute suicide tendency<br>-Severe comorbidities affecting life expectancy, medication, or QoL<br>-Severe fatigue<br>-Prominent impulse control disorder or dopamine dysregulation syndrome<br>-Acute psychosis or psychotic episode in the last 6 months<br>-Dementia medication<br>-Participation in other treatment studies within the last 2 months<br>-DBS<br>-Pregnancy or nursing period	**NEUROvitalis training:** n=33, 24 males (72.7%), 67.70 (7.19)<br>**Physical training:** n=31, 16 males (51.6%), 67.52 (8.32).	**NEUROvitalis training:**<br>-H&Y stage: range 1-4<br>-UPDRS-III: 24.00 (6.00-63.00)<br>**Physical training:**<br>-H&Y stage: range 1-3<br>-UPDRS-III: 25.00 (4.00-56.00)
**23**	**Reuter et al., 2012**	Germany	-Diagnosis of PD<br>-Presence of PD-MCI	-Severe concomitant diseases, which limit physical performance<br>-Second neurodegenerative disease<br>-Presence of dementia	**Cognitive, transfer and psychomotor training:** n=76, 40 males (52.63%)	**Cognitive, transfer and psychomotor training:**<br>-H&Y stage: range 2-4<br>**Transfer and cognitive training:**

ID	Source	Country	Inclusion Criteria	Exclusion Criteria	Sample (N, Gender, Age)	PD Stage (M, SD/N, %)
				-DBS	**Transfer and cognitive training:** n=75, 39 males (52%)<br>**Only cognitive training:** n=71, 36 males (50.7%)	-H&Y stage: range 2-4<br>**Only cognitive training:**<br>-H&Y stage 2-4
**24**	**Prats Paris et al., 2011**	Spain	-Diagnosis of PD (UK Parkinson's Disease Brain Bank Criteria)<br>-Age: 50–80 years<br>-H&Y stages 1-3<br>-Possible presence of PD-MCI	-MMSE <23<br>-Vocabulary subtest of WAIS-III <40<br>-GDS-15 >10<br>-On cholinesterase inhibitors<br>-Changes in their medication during the study<br>-Severe auditory or visual deficits or another psychiatric/neurological condition	**Cognitive training:** n=16, 7 males (43.75%), 64.75 (9.19)<br>**Speech training:** n=12, 7 males (58.33%), 65.42 (9.60)	**Cognitive training:**<br>-H&Y stage: 2.37 (0.76)<br>**Speech training:**<br>-H&Y stage: 2.25 (0.78)
**25**	**Sarasso et al., 2021**	Italy	-Diagnosis of PD PIGD phenotype<br>-H&Y stage ≤4<br>-Possible presence of PD-MCI<br>-MMSE ≥24	-Medical illnesses or substance abuse that could interfere with cognition<br>-Other major systemic, psychiatric, neurological, visual, and musculoskeletal disturbances or other causes of walking inability<br>-Contraindications to undergo MRI examination<br>-Brain damage at routine MRI, including lacunae and extensive cerebrovascular disorders	**DUAL-TASK + AOT-MI training**: n=13, 8 males (61.54%), 67.51 (6.12)<br>**DUAL-TASK training:** n=12, 8 males (66.67%), 63.81 (9.23)	**DUAL-TASK + AOT-MI training:**<br>-H&Y stage: range 2-3<br>-UPDRS-II: 10.38 (5.55)<br>**DUAL-TASK training:**<br>-H&Y stage: range 2-3<br>-UPDRS-II: 12.58 (5.14)
**26**	**Agosta et al., 2017**	Italy	-Diagnosis of PD<br>-H&Y stage <4<br>-Occurrence of FoG (FoG-Q)<br>-At least two of the following: observation of FoG by an experienced neurologist, the	-Medical illnesses or substance abuse that could interfere with cognition<br>-Other major systemic, psychiatric or neurological	**AOT group:** n=12, 10 males (83.33%), 69.0 (8.0)	**AOT group:**<br>-H&Y stage: 2.3 (0.4)<br>-UPDRS-III: 27.6 (9.7)<br>**Landscape group:**<br>-H&Y stage: 2.2 (0.3)

ID	Source	Country	Inclusion Criteria	Exclusion Criteria	Sample (N, Gender, Age)	PD Stage (M, SD/N, %)
			participant's verbal account of whether he/she had experienced FoG, the recognition of typical FoG in the patient's experience when this was identified and described to him/her by a physician<br>-MMSE>24<br>-PD duration: ≥5 years	illnesses (including musculoskeletal and visual disturbances)<br>-Other causes of gait impairment such as severe arthrosis or neuropathy<br>-Brain damage at routine MRI, including lacunae and extensive cerebrovascular disorders	**Landscape group:** n=13, 8 males (61.54%), 64.0 (7.0)	-UPDRS-III: 23.5 (7.9)
**27**	**Suarez-Garcia et al., 2021**	Colombia	Diagnosis of PD (UK Parkinson's Disease Brain Bank Criteria)	Not specified	**atDCS group:** n=11, 8 males (72.73%), 62.82 (7.49)<br>**stDCS group:** n=11, 9 males (81.82%), 66.45 (5.69)	**atDCS group:**<br>-H&Y stage: 1.9 (0.30)<br>-UPDRS-III: 18.64 (7.35)<br>**stDCS group:**<br>-H&Y stage: 1.09 (0.30)<br>-UPDRS-III: 19.45 (9.47)
**28**	**Vriend et al., 2021**	Netherlands	-Diagnosis of mild to moderately advanced idiopathic PD<br>-H&Y<4<br>-PD Cognitive Functional Rating Scale score >3<br>-Possible presence of PD-MCI	-MoCA <22<br>-Indications of current drug or alcohol abuse<br>-Moderate to severe depressive symptoms<br>-Impulse control disorder<br>-Non-benign psychotic symptoms<br>-History of traumatic brain injury	**Computerised cognitive training:** n=40, 20 males (50%), 63.3 (8.1)<br>**Computerised active control group:** n=39, 26 males (67.7%), 63.3 (6.4)	**Computerised cognitive training:**<br>-UPDRS-III: 20.6 (8.7)<br>-H&Y stage: range 1-3<br>**Computerised active control group:**<br>-UPDRS-III: 20.3 (9.4)<br>-H&Y stage: range 1-3

Abbreviations in alphabetical order:
ADL: Activities of Daily Living; **AOT:** Action Observation Training; **AOT-MI:** Action Observation Training-Motor Imagery; **atDCS:** anodal transcranial Direct Current Stimulation; **BDI-II:** Beck Depression Inventory-II; **CAGE-AID:** CAGE Adapted to Include Drugs Questionnaire; **CoRe:** Cognitive Rehabilitation computer software; **DBS:** Deep Brain Stimulation; **DSM-IV-TR:** Diagnostic and Statistical Manual of mental disorders-IV-Text Revision; **EF:** Executive Functions; **FoG:** Freezing of Gait; **FoG-Q**: Freezing of Gait Questionnaire; **GDS-15:** Geriatric Depression Scale-15; **H&Y:** Hoehn and Yahr Scale; **M:** mean; **MDS:** Movement Disorder Society; **MMSE:** Mini Mental State Examination; **MoCA:** Montreal Cognitive Assessment; **MRI:** Magnetic Resonance Imaging; **N:** number of participants; **NPI-Q:** Neuropsychiatric Inventory Questionnaire; **PD:** Parkinson's disease; **PD-CFRS:** Parkinson's disease Cognitive Functional Rating Scale; **PD-D:** Parkinson's disease-Dementia; **PD-MCI:** Parkinson's disease-Mild Cognitive Impairment; **PIGD:** Postural Instability and Gait Disorder; **QoL:** Quality of Life; **REHACOP:** cognitive rehabilitation program in psychosis; **ReSET:** Strategic Executive Treatment; **SCI questionnaire:** Subjective Cognitive Impairment questionnaire; **SD:** standard deviation; **stDCS:** sham transcranial Direct Current Stimulation; **tDCS:** transcranial Direct Current Stimulation; **UK:** United Kingdom;

UPDRS-II: Unified Parkinson's disease Rating Scale-II; **UPDRS-III:** Unified Parkinson's disease Rating Scale-III; **VR:** Virtual Reality; **WAIS-III:** Wechsler Adult Intelligence Scale-III
